# Metabolomics Coupled with Transcriptomics Approach Deciphering Age Relevance in Sepsis

**DOI:** 10.14336/AD.2018.1027

**Published:** 2019-08-01

**Authors:** Dingqiao Xu, Shanting Liao, Pei Li, Qian Zhang, Yan Lv, Xiaowei Fu, Minghua Yang, Junsong Wang, Lingyi Kong

**Affiliations:** ^1^Jiangsu Key Laboratory of Bioactive Natural Product Research and State Key Laboratory of Natural Medicines, School of Traditional Chinese Pharmacy, China Pharmaceutical University, Nanjing, China; ^2^Center for Molecular Metabolism, Nanjing University of Science and Technology, Nanjing, China.

**Keywords:** sepsis, biomarker, aging, transcriptomics, metabolomics

## Abstract

Sepsis is a severe disease frequently occurred in the Intenisive Care Unit (ICU), which has a very high morbidity and mortality, especially in patients aged over 65 years. Owing to the aging effect and the ensuing deterioration of body function, the elder patients may have atypical responses to sepsis. Diagnosis and pathogenesis of sepsis in this population are thus difficult, which hindered effective treatment and management in clinic. To investigated age effects on sepsis, 158 elderly septic patients and 71 non-septic elderly participants were enrolled, and their plasma samples were collected for transcriptomics (RNA-seq) and metabolomics (NMR and GC-MS) analyses, which are both increasingly being utilized to discover key molecular changes and potential biomarkers for various diseases. Protein-protein interaction (PPI) analysis was subsequently performed to assist cross-platform integration. Real time polymerase chain reaction (RT-PCR) was used for validation of RNA-seq results. For further understanding of the mechanisms, cecal ligation and puncture (CLP) experiment was performed both in young and middle-aged rats, which were subjected to NMR-based metabolomics study and validated for several key inflammation pathways by western blot. Comprehensive analysis of data from the two omics approaches provides a systematic perspective on dysregulated pathways that could facilitate the development of therapy and biomarkers for elderly sepsis. Additionally, the metabolites of lactate, arginine, histamine, tyrosine, glutamate and glucose were shown to be highly specific and sensitive in distinguishing septic patients from healthy controls. Significant increases of arginine, trimethylamine N-oxide and allantoin characterized elderly patient incurred sepsis. Further analytical and biological validations in different subpopulations of septic patients should be carried out, allowing accurate diagnostics and precise treatment of sepsis in clinic.

As a common cause of death in hospitalized patients worldwide, sepsis is associated with high morbidity and mortality despite continual efforts toward its diagnosis and treatment [1]. The elder are known to be in a state of immunosenescence, whose protective immunity is weak to elicit an effective response to invading organisms than the youth [2-4]. Therefore, sepsis is increasingly known as a disease of the aged, and the number of elderly patients (defined by World Health Organization as ≥65 years old) with severe sepsis and septic shock has been in steadily increasing [5, 6]. Invasive devices and interventions are less effective in elderly patients than in younger patients in clinical practice [7]. In addition, aged patients with deteriorated immunity are especially vulnerable to various sepsis associated comorbidities. Therefore, increasing interest has been put to the pathogenesis of sepsis in the elderly during disease progression to improve diagnostic sensitivity and therapeutic outcomes for the elderly in clinical settings.

Metabolic profiling, or metabolomics, provides a wealth of data on metabolic alterations that reflect genetic, epigenetic, and environmental factors influencing cellular physiology [8]. As the results of down-stream life activities, metabolome is sensitive to disease and other stimuli, and close to phenotype, which make metabolomics applicable for disease diagnosis and treatment assessment [9]. Gene expression profiling, also called transcriptomics, is capable of surveying the entire genome and identifying novel candidate pathways and targets, thus yielding further insight into disease including sepsis [10, 11]. Transcriptomics has been successfully applied to uncover potential biomarkers for early diagnosis and risk assessment of sepsis and identifying dysregulated pathways and transcriptional programs related to sepsis [12]. Integration of transcriptomics and metabolomics may yield even greater in-depth insight into the pathogenesis of sepsis than either approach alone, help mapping metabolites and genes to relevant biochemical pathways.

In this study, metabolomic and transcriptomic approaches were first used to identify differential metabolites and genes related to in the elderly sepsis patients (ESEP). To explore further the age factor on the outcome of sepsis, young and middle-aged rats were subjected to cecal ligation and puncture (CLP), and integrated metabolomic approach and molecular biology was used to identify the differences. Through cross-platform integration, protein-protein interaction (PPI) analysis of metabolomic and transcriptomic data has identified a number of metabolites and signaling pathways that are relevant to age factor in sepsis and important in its pathophysiology.

**Figure 1.**
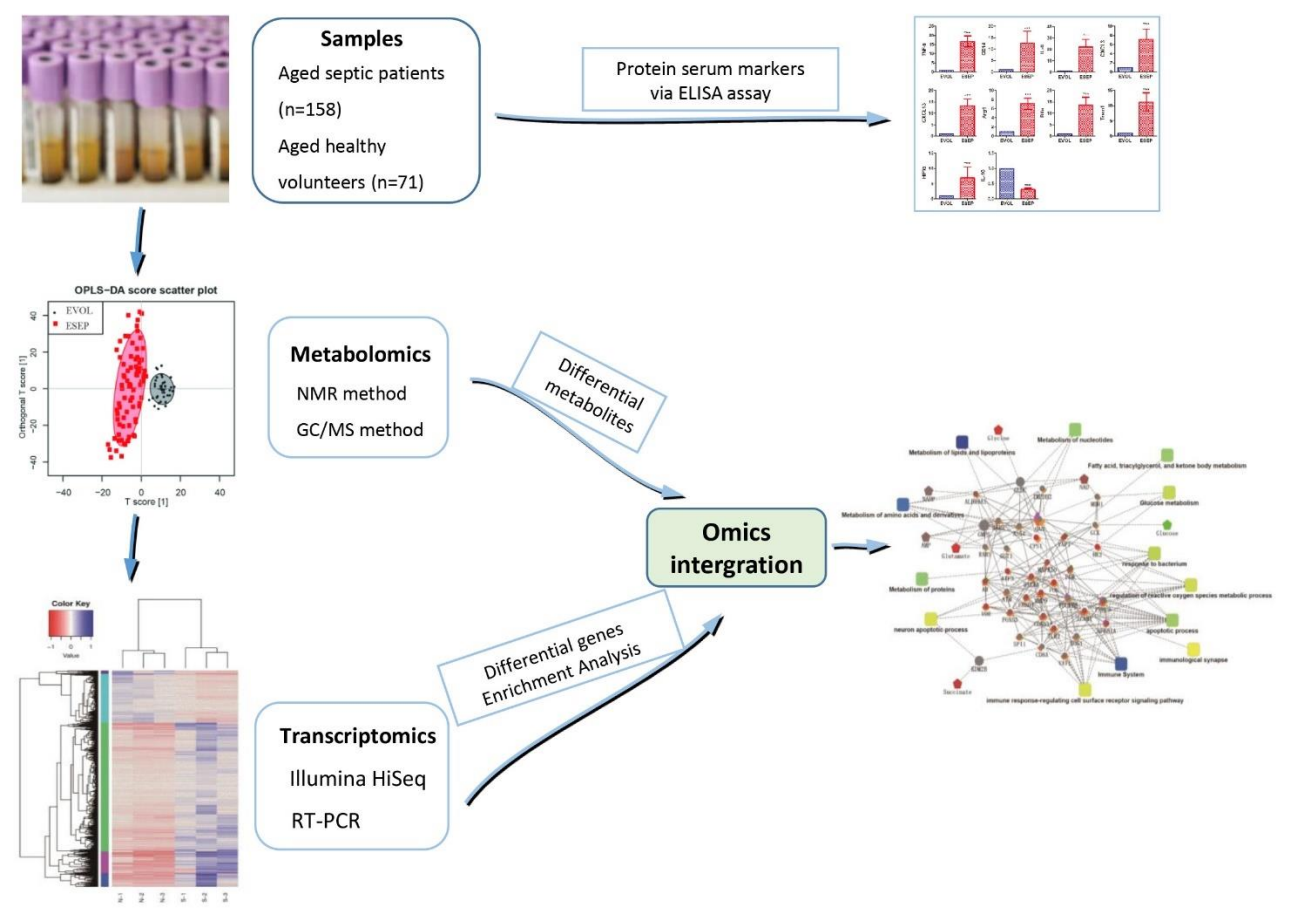
An overview workflow of the comprehensive analysis of metabolomics and transcriptomics in sepsis.

## MATERIALS AND METHODS

An overview of the workflow for the integrated metabolomic and transcriptomic approach to study sepsis is presented in Fig. 1.

### Chemicals and reagents

Sodium 3-trimethylsilyl-1-(2, 2, 3, 3-2H4) propionate (TSP) was obtained from Sigma (St. Louis, Mo, USA). Deuterium oxide (D2O, 99.9 %) was purchased from Sea Sky Bio Technology Co. Ltd. (Beijing, China). Pentobarbital and methanol were obtained from Sinopharm Chemical Reagent Co., Ltd. (Shanghai, China). Heptadecanoic acid, methoxyamine hydrochloride and N-methyl-N-trimethylsilyl-trifluoroacetamide (MSTFA) with 1% trimethyl-chlorosilane (TMCS) for derivation was purchased from Sigma-Aldrich (St. Louis, MO). Ultra-pure distilled water, prepared from a Milli-Q purification system, was used. The kits of superoxide dismutase (SOD), nitric oxide (NO), glutathione (GSH), creatine, acetylcholin esterase (AchE) and choline acetyltransferase (ChAT) were bought from Nanjing Jiancheng Bioengineering Institute (Nanjing, China). The Enzyme-linked immunosorbent assay and microarray immunoassay (ELISA) kits of lactic dehydrogenase (LDH), aspartate aminotransferase (AST) and alanine aminotransferase (ALT) were obtained from Senbeijia Bioengineering Institute (Nanjing, China). The other ELISA kits of interleukin-6 (IL-6), monocyte chemotactic protein 1 (MCP-1), CX3C chemokine receptor 1 (CX3CR1), macrophage inflammatory protein-1α (MIP-1α) and recombination activating gene 1 (RAG-1) were obtained from R&D systems (Minneapolis, MN, USA). Primary antibodies of toll-like receptor4 (TLR4), myeloid-differentiation factor88 (MyD88), tumor necrosis factor receptor associated factor6 (TRAF6) and Arginase-1 (Arg1) were purchased from Gene Tex Inc. (United States). Primary antibodies of high mobility group box1 (HMGB-1), phosphorylation of IKKα/β (p-IKKα/β), phosphorylation of p65 (p-p65), phosphorylation of IκBα (p-IκBα), Cyclooxygenase-2 (COX-2), phosphorylation of tyrosine hydroxylase (p-tyrosine), phosphorylation of mitogen-activated protein kinases (p-ERK, p-JNK, p-p38), Kelch-like ECH-associated protein 1 (Keap1), Histone H3, c-Jun, c-Fos, cleaved-PARP antibodies and inducible nitric oxide synthase (iNOS) were purchased from Cell Signaling Technology (Danvers, MA). Primary antibody against CD14 was purchased from EnoGene Biotechnology (Nanjing, China). Antibodies of nuclear factor-like2 (Nrf2) and Heme Oxygenase 1(HO-1) were purchased from Abcam plc. (Abcam, Cambridge, UK). Anti-rabbit IgG antibody, anti-mouse IgG antibody were also purchased from Cell Signaling Technology, which were used as secondary antibodies. β-actin antibody was purchased from Sigma-Aldrich (St. Louis, MO).

### Animals and animal experiment design

One-year-old male specific-pathogen-free (SPF) rats (480-500 g) and 6-week-old male Sprague-Dawley rats (180-220 g) were obtained from the Experimental Animal Center of Yangzhou University (Yangzhou, China). The animals were acclimated for 10 days prior to surgery. All animals were reared and handled in strict accordance with the requirements of the Animal Ethics Committee of China Pharmaceutical University and the guidelines for the care and use of laboratory animal from the National Institute of Health (license number: SYXK (Su) 2017-0011).

The animals were assigned to the following four groups (n = 15 per group): (1) vehicle-treated young sham (YSham), (2) vehicle-treated middle-aged sham (ESham), (3) vehicle-treated young CLP (YCLP), (4) vehicle-treated middle-aged CLP (ECLP). Polymicrobial sepsis caused by CLP was induced as described previously [13]. The details of the methods of animal model are described in Supplemental Material. A day after the operation, rats were anesthetized with pentobarbital, and blood samples were isolated from the ocular vein and collected in EDTA tubes. The whole blood was centrifuged at 3,000 *g*, and the plasma was collected. The liver tissues of middle-aged and young rats were harvested under aseptic conditions at 24 h after the operation. The plasma and tissue samples were frozen immediately in liquid nitrogen and stored at -80 °C until analysis. The tissues were used for Western blot and histopathology testing.

### Patient selection and clinical data collection

Sepsis was identified according to international guidelines in addition to the presence of at least two of the following four clinical criteria: (a) fever or hypothermia (temperature of >38 °C or <36 °C), (b) tachycardia (>90 beats/min), (c) tachypnea (> 20 breaths/min) or the need for mechanical ventilation, and (d) an altered white blood cell count above 12,000 cells/μL or below 4,000 cells/μL [1]. The inclusion criteria required microbiological pathology results ensuring bacterial infection and consensus by the consulting physician [14]. In total, 86 septic patients, aged 65 to 82 years, were enrolled in this study. The work was conducted in accordance with the Declaration of Helsinki and the International Conference on Harmonization-Good Clinical Practices (ICH-GCP). This study was approved by the Institutional Review Board of the Huangshi Central Hospital, Hubei Province, accredited by Joint Commission International (JCI).

**Figure 2.**
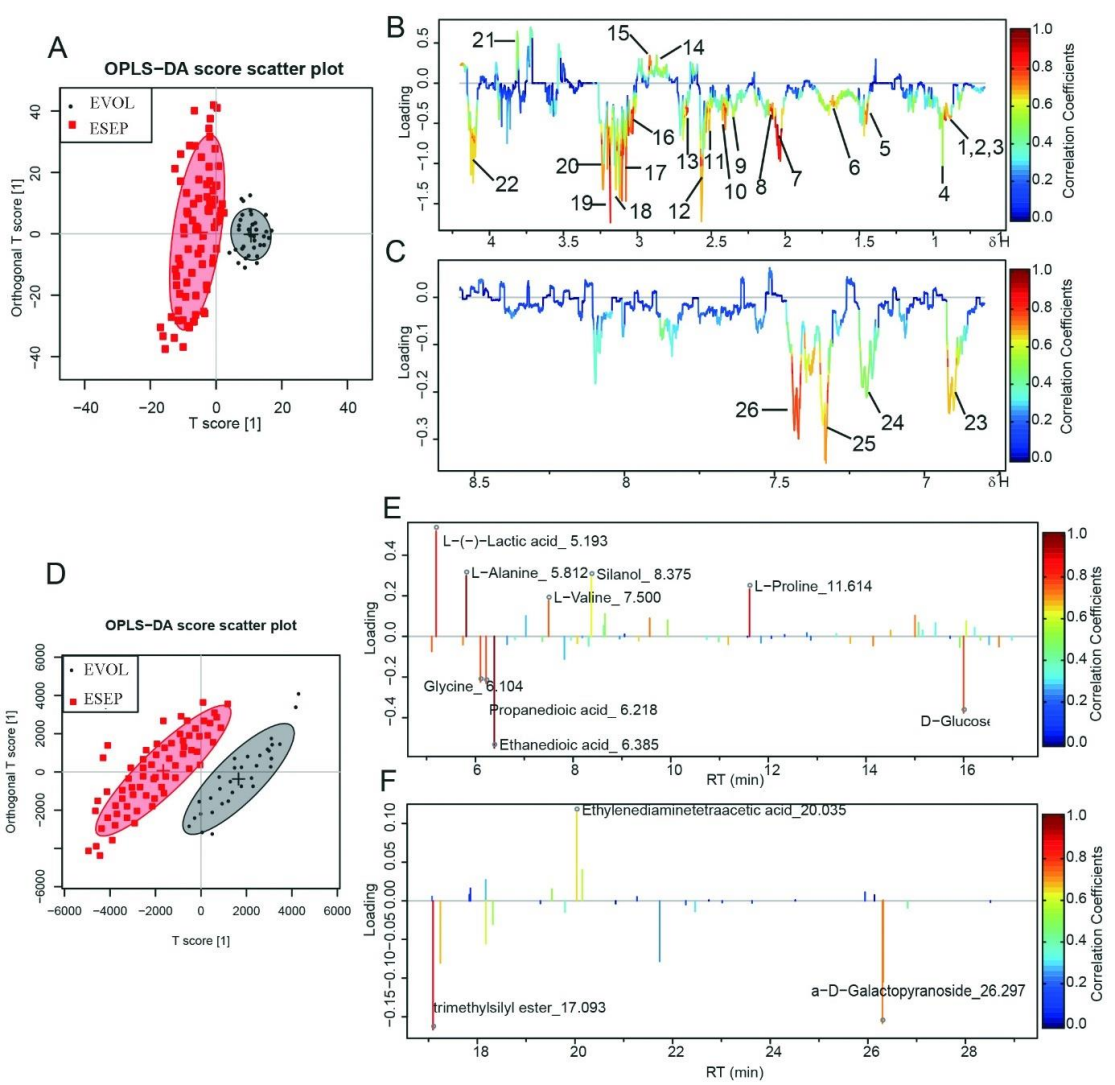
OPLS-DA analysis of metabolic profiles between ESEP and EVOL groups for plasma Score plots (**A**) and color-coded coefficient loadings plots (**B, C**) for the plasma of septic patients based on ^1^H NMR analysis; Score plots (**D**) and color-coded coefficient loadings plots (**E, F**) for the plasma of septic patients based on GC-MS analysis. Significantly changed metabolites were assigned in the loading’s plots. Downward and upward peaks represent increased and decreased concentrations in pathogenic group. Symbols of ? (black filled circles), ¦ (red filled squares) represent the control and pathogenic groups, respectively.

### Baseline characteristics of patients

Diagnosis of sepsis syndrome was based on fever, infectious focus or positive blood culture, leukocytosis or leukocytopenia, and thrombocytopenia or a decrease of more than 30% in thrombocyte count [15]. Samples were obtained at one-day intervals after the diagnosis of sepsis syndrome. The control donors were apparently healthy volunteers (EVOL) recruited among laboratory personnel and the relatives of patients (n = 39; average age, 66 ± 10 years). After written consent was obtained, blood samples were collected in the morning from all the subjects stored in Na_2_ EDTA vacutainer tubes and immediately refrigerated (4 °C). Within 2 h of collection, they were centrifuged at 4,000 *g* for 10 min at 4 °C. The plasma was transferred into new vials and immediately stored frozen (-80 °C) until sample preparation. The sample pre-treatment methods for ^1^H NMR and GC-MS were detailed in the methods provided in Supplemental Materials.

### Hematoxylin and eosin (H&E) staining of specimens from rats

For histological evaluation, organs were quickly removed, immersed in 10% neutral-buffered formaldehyde for 24 hours, and then embedded in paraffin and sliced into 5 µm thick sections. The sliced sections were stained with hematoxylin and eosin (H&E) and scoring for histopathological change was performed under a light microscope (Optithot; Nikon) with a digital camera (DXM1200; Nikon). According to the degree of each viscera lesion from mild to serious, each parameter was graded on a scale of 0-3, with 0 meaning “absent”, with 1 meaning “soft”, with 2 meaning “moderate”, with 3 meaning “severe”. The total injury score was expressed the sum for all parameters. The total injury score was expressed as the sum of all parameters [16].

### Total RNA extraction and quantification

Fasting whole blood samples were collected in the morning from six subjects (three ESEP and three EVOL). The blood samples were immediately frozen in liquid nitrogen and stored at -80 °C until later use for total RNA isolation. In an RNase-free environment, total RNA was extracted from human blood using TRIzol Reagent (Invitrogen) according to the manufacturer’s recommendations, and a SpectraMax Plus 384 enzyme-labeling instrument (Molecular Devices, Sunnyvale, USA) and 1% agarose gels were used to determine the quantity and quality of the RNA [17].

### cDNA library construction and transcriptome sequence processing and assembly

According to the manufacturer’s recommendations, a cDNA library was prepared with a kit provided by Illumina. Total RNA was extracted from the human blood samples. Then, poly (A) mRNA was purified from the total RNA using oligo (dT) beads. After purification, the mRNA was sheared into small pieces using fragmentation buffer. First-strand cDNA was annealed with random primers, using cleaved mRNA fragments as templates. The second-strand cDNA was synthesized and purified immediately and ligated to index adapters. Finally, the cDNA library was constructed and subjected to high-throughput sequencing with an Illumina HiSeq 2500 system. The raw data were translated into FASTQ format and then compressed into.gz files to be deposited in the National Center for Biotechnology Information database under Bio Project accession number PRJNA422660. Clean reads of RNA nucleotide sequences were generated using TopHat software. Differential gene expression analysis was performed by using the DESeq Bioconductor package with a false discovery rate (FDR) of <0.05, with the threshold of significance set to a relative fold change >2. Gene Ontology (GO) and KEGG (Kyoto Encyclopedia of Genes and Genomes) enrichment were analyzed to detect the functions and pathways of differentially expressed genes (DEGs) [18]. Three technical replicates were conducted for each sample.

### Quantification of gene expressions by real-time RT-PCR.

Real-time RT-PCR was performed to validate the results derived from RNA-seq. Total RNA from the blood of middle-aged and young rats and that of humans was isolated using TRIzol Reagent (Invitrogen). In addition, the quantity and quality of the RNA were determined using a SpectraMax Plus 384 enzyme-labeling instrument. The details of the methods are described in Supplemental Material.

### Western blot analysis

For protein analysis, liver tissues were removed from middle-aged and young rats and frozen until use. The details of the methods are described in Supplemental Material.

### Measurement of clinical biochemistry and cytokines

The serum levels of SOD, NO, GSH, GSSG and creatine in patients, middle-aged and young rats were assayed with commercially available kits, as were the plasma levels of AchE and ChAT, while the serum levels of ALT, AST, IL-6, IL-1β, LDH, MCP-1, CX3CR1, MIP-1α and RAG-1 were measured with ELISA kits according to the manufacturer's instructions. All samples were assayed in triplicate. All laboratory personnel were blinded to the clinical information of the patients.

### ANOVA-simultaneous component analysis (ASCA)

Moreover, in order to investigate the interaction between age and CLP, we applied the ANOVA-simultaneous component analysis (ASCA) method for the effect of age or/and CLP on the metabolism [19]. ASCA was first applied to split data according to the experimental factors, namely age, CLP, and the age × CLP interaction.

### Statistical analysis

Assays were conducted at least three times unless otherwise stated. All the experimental data except for mortality were expressed as the means ± standard deviation (SD). Survival data were analyzed by Kaplan-Meier curves and the log-rank test. Western blotting data were analyzed by two-way analysis of variance, and the Bonferroni test was used for *post hoc* comparisons. Differences between the groups were considered statistically significant at *p* < 0.05. Results were presented as the mean ± SD. The multiomics data analysis tool OmicsBean (www.omicsbean.com) was used to analyze the obtained metabolomics and transcriptomics data, and the metabolites and genes were classified according to their biological functions, subcellular locations and molecular functions based on Gene Ontology (GO) categories. Protein-protein interaction (PPI) analysis was performed using Cytoscape software, in which a confidence cutoff of 400 was used: interactions with confidence scores greater than 400 were shown as solid lines between genes/proteins, and the remaining interactions were shown as dashed lines.

**Figure 3.**
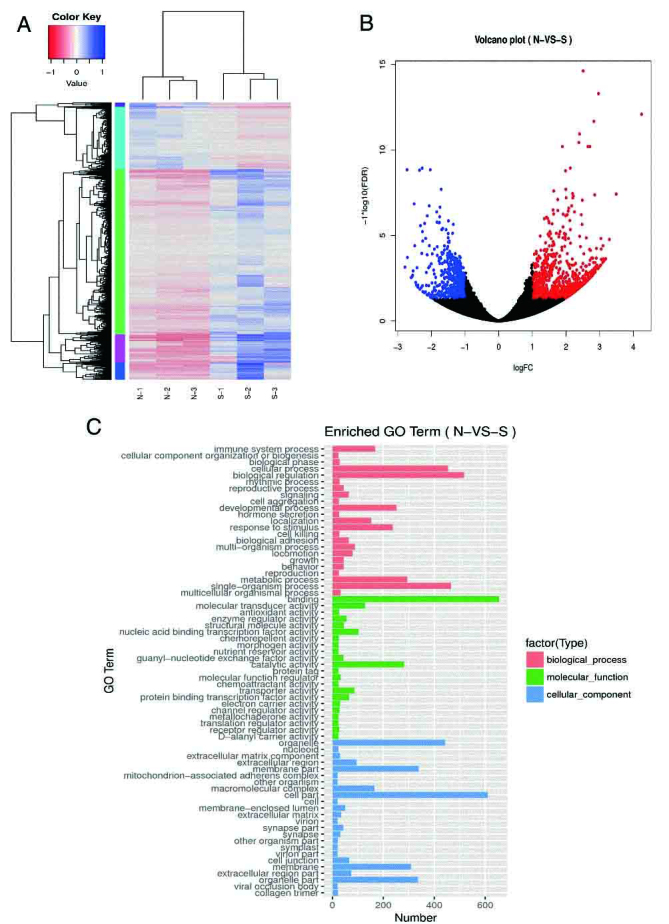
Plasma transcriptomics response caused by sepsis in elderly patients (**A**) Heat map of differentially expressed genes (DEGs) identified by RNA-seq between groups (P <0.05). Hierarchical clustering of DEGs in EVOL samples (N-1, N-2, and N-3) compared with the ESEP samples (S-1, S-2, and S-3). (**B**) Volcano plots of DEGs. A total of 1636 DEGs, including 1088 up-regulated and 548 down-regulated genes, threshold of significance as fold change was >2, FDR <0.05. (**C**) Histogram diagram of Gene Ontology (GO) classification. The results of DEGs are summarized in three major categories: biological process (red), molecular function (green), and cellular component (blue). The y-axis on the left indicates the enriched GO terms; the x-axis indicates the number of DEGs.

**Table 1:** Identified metabolites with fold changes between groups and p-values in rat plasma.

No.	Metabolites	ECLP *vs* ESham		YCLP *vs* YSham		ECLP* vs* YSham		ECLP *vs* YCLP	

		FC	P	FC	P	FC	P	FC	P
1	Isoleucine	1.21		1.07		0.92		1.11	
2	Leucine	1.22		1.04		1.07		1.31	
3	Valine	1.29		1.26		0.84		1.16	
4	Hydroxybutyrate	3.6	**	2.76	*	0.32	*	1.89	*
5	Lactate	1.37		1.29		0.72	*	1.14	
6	Alanine	1.65	*	0.86		1.39		1.2	
7	Allantoin	1.55	**	0.68	**	1.9	***	1.29	*
8	Acetate	1.16		1.24		0.93		1.76	**
9	NAA	1.05		1.05		1.08		1.13	
10	Methionine	1.02		1.28	*	0.9		1.15	
11	Glutamate	2.14	*	1.18		1.11		1.31	
12	Glutathione	0.96		1.06		1.21		1.28	
13	Succinate	1.77	*	1.57	*	0.59	*	1.12	
14	Glutamine	1.07		1.39	*	1.03		1.43	*
15	Arigine	1.79	*	0.9		1.28		1.33	
16	Creatine.Pcr	1.53	*	1.38		0.85		1.78	**
17	Ornithine	1.58	*	1.29		0.84		1.08	
18	Ethanolamine	1.27		0.92		1.11		1.01	
19	Choline	2.27	*	1.14		0.76		1.86	**
20	Choline. /O.Phosphocholine	2.53	*	1.37		0.6	*	1.82	*
21	Glucose	0.61	*	1.21		1.21		0.38	**
22	Taurine	0.72		0.96		0.99		0.95	
23	Betaine	0.93		1		1		1	
24	Glycine	1.69	*	0.94		0.91		0.85	
25	Maltose	1.23		1.25		0.72		0.9	
26	Ascorbate	0.94		1.01		0.95		0.96	
27	Inosine	1.31		1.04		0.95		0.99	
28	Uridine	1.14		1.13		0.84		0.95	
29	TMAO	1.25	*	1.11		1.12		1.65	***
30	Fumarate	1.3		0.88		0.99		0.87	
31	Tyrosine	1.05		0.96		1.58	*	1.51	*
32	Histamine	2.06	*	0.82		1.62	*	1.32	
33	Tryptophan	0.91		0.99		1.23		1.22	
34	Phenylalanine	1.36		1.62	*	0.8		1.3	
35	Cytidine	1.09		1.17		0.96		1.12	
36	Methylxanthine	1.11		1.45	*	0.92		1.33	
37	Formate	0.71		0.94		1.38		1.3	

FC: Fold Change; P: P-value; ECLP: Middle-aged CLP rats; ESham: Middle-aged Sham rats; YCLP: Young CLP rats; YSham: Young Sham rats. Color-coded according to the fold change value, red represents increased and blue represents decreased concentrations of metabolites. P-values corrected by BH (Benjamini Hochberg) methods were calculated based on a parametric Student’s *t*-test or a nonparametric Mann-Whitney test (dependent on the conformity to normal distribution).

* *p*<0.05,

** *p*<0.01,

*** *p*<0.001.

## RESULTS

Clinical characteristics of ESEP and EVOL were summarized in Supplementary Table 1.

### Histological analysis

Histological examination revealed cell structure in the liver tissues of ESham animals. Increased inflammatory cell infiltration, as well as moderate portal inflammation and hepatocellular necrosis, was found in the liver sections of ECLP rats. The results of the survival experiments are shown in Supplementary Figure 1F.

**Figure 4.**
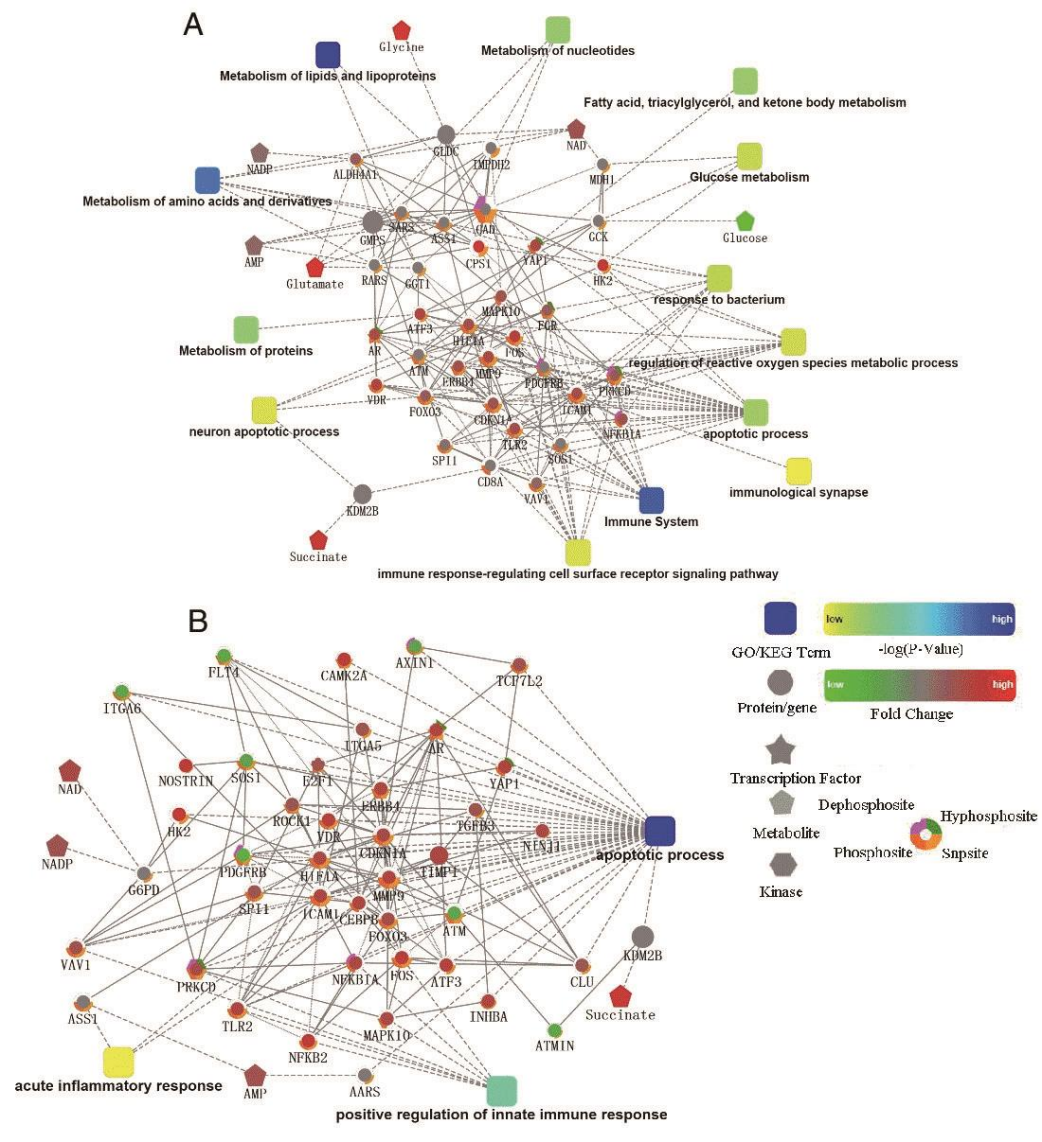
A network of protein-protein interaction (PPI) The PPI analysis was based on fold change of gene/protein, protein-protein interaction, KEGG pathway enrichment and biological process enrichment. Circle nodes refer to genes/proteins. Rectangle refers to KEGG pathway or biological process, which was filled with color gradient from yellow (low *p*-value) to blue (high *p*-value).

**Figure 5.**
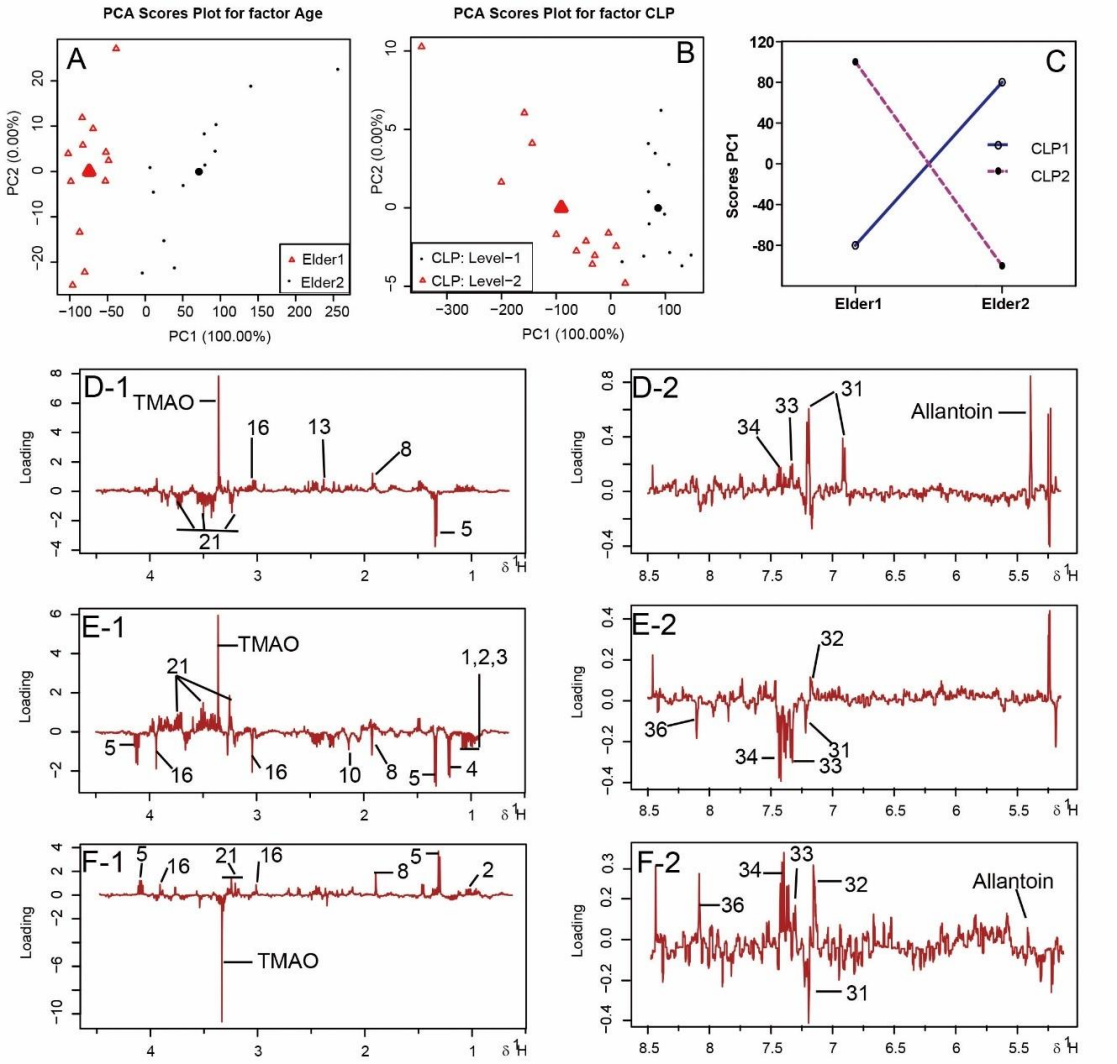
Scores on the first and second principal components and average scores for the factor Age (A) and CLP (B) Interaction ‘Age × CLP’ model (**C**) scores on the first principal component of the corresponding submodels. The loadings (**D**, **E**, and **F**) belonging to the first component for the factor Age, CLP and the interaction ‘Age × CLP’.

### Metabolomic analysis of the plasma of aged septic patients

To understand the plasma metabolite signatures of ESEP group, we identified 26 metabolites (included in 37 metabolites identified in septic rats) by NMR (Supplementary Fig. 2 and Supplementary Table 2), which were also confirmed by GC-MS methods. In the Orthogonal partial least square-discriminate analysis (OPLS-DA) score plots, the EVOL and ESEP groups (Fig. 2A) were satisfactorily separated in the score plots of ^1^H NMR and GC/MS (Fig. 2D). The color-coded loading plots for OPLS-DA revealed differences in plasma extract metabolite profiles between the EVOL and ESEP groups (Fig. 2B, 2C for ^1^H NMR analysis, and Fig. 2E, 2F for GC/MS analysis).

### Metabolomic analysis of the plasma of middle-aged and young adult rats

YSham, YCLP, ESham, and ECLP groups exhibited a clear separation in the OPLS-DA score plots (Supplementary Fig. 3A) of plasma extracts. The color-coded loading plots (Supplementary Fig. 3E) revealed the differential plasma metabolites (Table 1) between groups. Comparisons of ECLP *vs.* YCLP and ESham *vs.* YSham were incorporated in the supporting information as Supplementary Fig. 3B, C and Fig. 3F, G, respectively.

**Figure 6.**
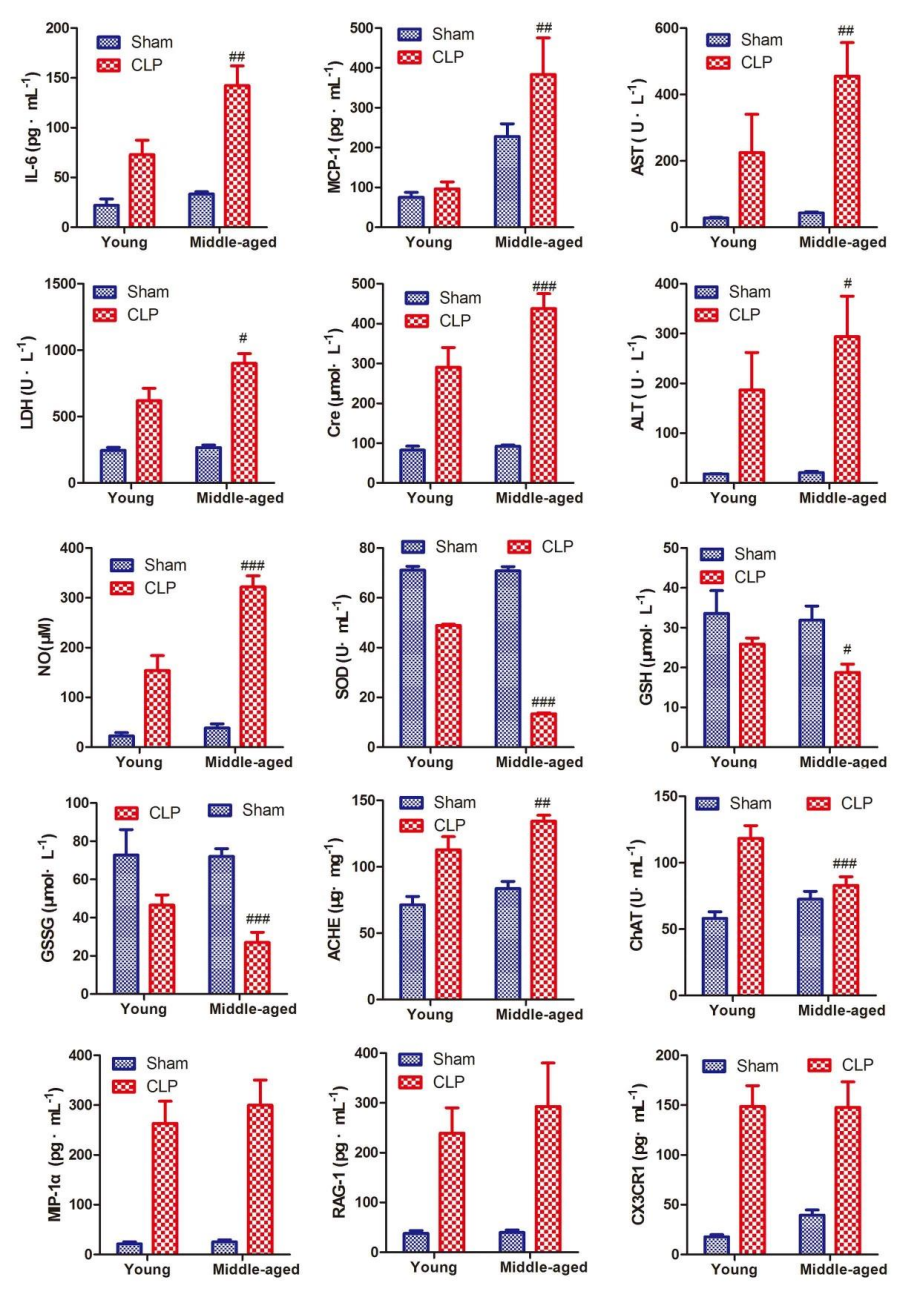
Biochemical parameters including inflammation markers and immune cytokines were determined in rats Histograms for clinical chemistry results of MCP-1, MIP-1α, RAG-1, LDH, IL-6, CX3CR1, ALT, AST, SOD, GSSG, GSH, NO and for creatine of serum in septic middle-aged (ECLP) and young rats (YCLP) (n=6). Data in serum are expressed as mean ± S.D. ^#^*p* < 0.05,^ ##^*p* < 0.01 and ^###^*p* < 0.005 for YCLP *vs.* ECLP group.

**Figure 7.**
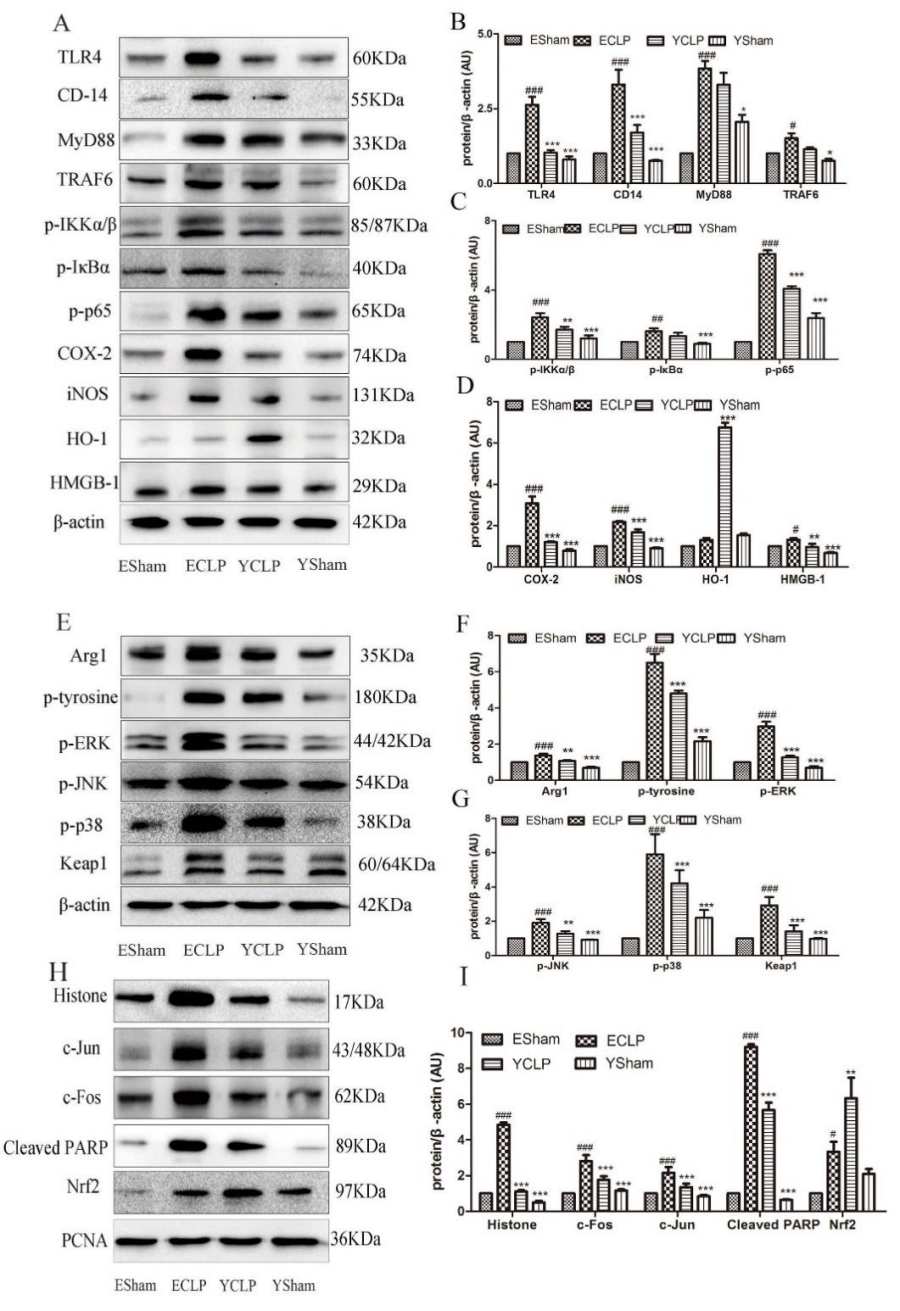
Aging effects on TLR4/NF-κB and MAPK signal pathway in rat livers (**A**) Aging effects on TLR4/ NF-κB signal pathway. The expression of proteins in TLR4/NF-κB were up-regulated in ECLP group (**B, C, D**). (**E**) Shown are western blots for MAPK signal pathway. The expression of proteins in MAPK were up-regulated in ECLP group (**F, G**). (**H**) Histone, c-Jun, c-Fos, Cleaved PARP and Nrf2 expression level were analyzed by western blotting. The accompanying bars represent intensity ratio of protein relative to β-actin. Results are expressed as mean ± SD. *p < 0.05, **p < 0.01 and ***p < 0.005 for YCLP (vehicle-treated young CLP) group vs. ECLP group. #p < 0.05, ##p < 0.01 and ###p < 0.005 for ESham (vehicle-treated sham) group vs. ECLP group. (n=6).

### Plasma transcriptomic response caused by sepsis in elderly patients

To trace variations in upstream of metabolites, we examined the transcriptome of ESEP and EVOL. Approximately 50,189,555 raw reads from each sample were obtained by RNA-Seq; and after quality filtration, ca. 91% of reads were mapped as clean reads, as shown in Supplementary Table 3. With the threshold of significance as relative fold change >2 and FDR <0.05, a total of 1636 DEGs were finally kept. GO and KEGG assignments were used to classify the genes affected by sepsis. In GO term analysis, a high percentage of genes were classified under the terms “metabolic process”, “binding”, “transporter activity” and “cell part” (Fig. 3C). The expression levels of genes involved in the cell surface and biosynthesis were significantly changed in the sepsis group. KEGG ontology assignments, determined through mapping with the KEGG database, identified 166 genes that were significantly enriched (p < 0.05), spanning 20 pathways (Supplementary Fig. 4).

### Integration analysis of plasma metabolomics and transcriptomics

PPI network analysis (Fig. 4) of the metabolomics and transcriptomics data was made using Cytoscape. Energy metabolism and amino acid metabolism, especially arginine metabolism, were markedly disturbed in sepsis (Fig. 4A). The ESEP group has significantly up-regulated gene expression in pathways involved in the proliferation of immune cells (Fig. 4B). Sepsis activated several important pathways, such as Toll-like receptor signaling, and the canonical NF-κB, immune and the MAPK signaling pathways. In addition, genes involved in the HIF-1 signaling pathway, TNFR2 signaling and the PI3K/AKT signaling pathway were preferentially up-regulated by sepsis (Supplementary Fig. 5).

### Analysis of age effects by ASCA

The age-related NMR spectral dataset was further analyzed by ASCA to evaluate the significance and variance distribution of age, CLP and their interaction terms. The percentage contribution of each effect to the sum of squares of the data matrix and the statistically significant values obtained from permutation tests in ASCA models are listed in Supplementary Table 4. The score plots of the first component for factors ‘age’, ‘CLP’ and their interaction are presented in Fig. 5A-C, respectively, with the corresponding loadings plots as Fig. 5D-F. The markedly varied metabolites for the first component of ASCA analysis are listed in Supplementary Table 5.

### Validation of inflammation markers and immune cytokines in the plasma of rats

Biochemical parameters including inflammation markers and immune cytokines were determined in patients and rats. The levels of IL-6, MCP-1, MIP-1α, RAG-1, LDH, ALT, AST, NO and creatine were significantly increased in ESEP group as compared with EVOL group (Supplementary Table 1). Similar results were found in sepsis models on ECLP rats. The trends for these parameters hold true for YCLP group, however, to a much less content (Fig. 6). Plasma AchE and ChAT activities showed no apparent changes in ESEP group (Supplementary Table 1) but were significantly enhanced in ECLP rats (Fig. 6).

### Validation of cytokine mRNA expression in the plasma of patients and rats

For validation of the RNA-Seq results, ten genes mostly changed were quantified in the plasma of patients and rats by real time RT-PCR. Among them, expressions of TNF-α, CD14, IL-6, CXCL13, Arg1, CXCL3, Btla, HIF1α and Trem1 mRNAs were significantly increased in sepsis groups, while that of IL-10 was markedly decreased, which were in consistent with the results of RT-PCR (Supplementary Fig. 6 for patients, and Supplementary Fig. 7 for rats).

### Validation of some key signaling pathways in the liver tissue of rats

Western blotting analyses of proteins belonging to sepsis related pathways such as Toll-like receptor, canonical NF-κB, immune and MAPK signaling were performed. Liver levels of TLR4, CD-14, MyD88, TARF6, p-IKKα/β, p-p65, p-IκBα, COX-2, iNOS, and HMGB-1 were significantly increased in CLP modeling groups with less extent in young rats (Fig. 7).

## DISCUSSION

In this study, metabolomics and transcriptomics were used to identify significant alterations of metabolite and gene transcription levels in ESEP group. These potential biomarkers and key signaling pathways were further validated by non-targeted metabolomics on ECLP rats. The most affected pathways were energy metabolism (glucose metabolism), amino acid metabolism (arginine and tyrosine), inflammation, and oxidative stress. Arginine, TMAO, and allantoin are the major metabolites associated with age related differences in sepsis.

### Energy metabolism

Sepsis is a major catabolic insult characterized by enhanced energy expenditure [20]. Fumarate and succinate are the critical intermediates of the TCA cycle [21]. Significant alterations of TCA cycle intermediates were found in the sepsis groups compared with the ESham group, with increased levels of fumarate and succinate, suggesting disturbance in energy metabolism in sepsis and consequent energy production inefficiency. Consequently, glucose was markedly decreased in the plasma of the ESEP and ECLP groups, reflecting its overconsumption as an energy supply, which was also supported by the increased level of xanthine in the ECLP, indicating a high ATP turnover rate [21]. The inhibition of the TCA cycle calls for the enhancement of another energy supply by anaerobic respiration, consisting of glycolysis followed by the conversion of pyruvate to lactate or alanine [22, 23]. On the basis of the transcriptomics results, we can easily infer that significant changes related to energy metabolism occurred in ESEP group. Acyl-CoA dehydrogenase family member 9 (ACAD9) and short/branched-chain acyl-CoA dehydrogenase (ACADSB), which encode enzymes responsible for catalyzing the reactions of acyl-CoA, were found to be down-regulated. In addition, down-regulation of the GLUT2 isoform promotes hypoglycemia in models of sepsis [24]. As a result, the rate of glucose utilization is enhanced in ESEP group. The enzymes of the glycolysis pathway are up-regulated during sepsis. These include lactate dehydrogenase (LDH), hexokinase 2 (HK2), fructose-2,6-bisphosphatase 2 (PFKFB2), and fructose-1,6-bisphosphatase 1 (FBP1). The enzyme pyruvate dehydrogenase phosphatase (PDP), which facilitates the conversion of pyruvate to acetyl-CoA to enter the aerobic citric acid cycle, is down-regulated in ESEP. Thus, pyruvate can continue forming lactate anaerobically during sepsis [25]. All these findings support our metabolomics study. Consistently, glycolysis was significantly increased in ECLP rats.

### Amino acid metabolism

Levels of most amino acids including arginine were greatly increased in ESEP group, characterized an accelerated catabolism of sepsis [26-28]. As an important regulatory factor in the availability and function of arginine [26], the activities of Arg-1 were greatly increased in ESEP group. Catalyzed by Arg-1, arginine was converted to ornithine. In ESEP group, plasma ornithine was significantly increased, which indicates enhanced conversion from arginine to ornithine. However, plasma arginine production showed no significant difference between YCLP and YSham controls, so did arginine is found in young adult septic patients and non-septic intensive care unit controls [26, 29]. The relationships of arginine accumulation with inflammatory responses are still in debate [30]. Our metabolomics and transcriptomics data suggested that elevated levels of arginine might be a clinical hallmark of sepsis in elderly patients.

### Inflammation

Sepsis is characterized by severe inflammatory responses to infection, the severity of which is closely related with sepsis-induced lethality [31]. TNF-α, a pro-inflammatory cytokine, is a dominant inflammatory mediator that mediates a multitude of inflammatory events; IL-6, IL-1β, MCP-1, MIP-1α, RAG-1, CXCL13, Arg1, CXCL3, CX3CR1, Btla, HIF1α and Trem1 are pleiotropic cytokines and immune factors that can amplify and catalyze the inflammatory response [32-34]. These inflammation markers and immune cytokines of ESEP group were markedly higher than those in EVOL group. Except for MIP-1α, RAG-1, and CX3CR1, most of these cytokines were significantly increased in ECLP group as compared with YCLP group. With the increase of age, inflammatory response to infection increase. However, such radical inflammatory reaction in the onset of sepsis is not good in the long run, which will ultimately impair the immune system of the body. The elderly is generally of weak immunity than the young. The over mobilization of the immune system further decreased the immunity of the elder, which will in the end produce a status of immunosuppression, aggravating sepsis (Fig. 6 and Supplementary Fig. 7).

As compared with the YCLP group, significant increases of choline, AchE and decrease of ChAT were observed in ECLP group. However, in transcriptomics results, the levels of AchE and ChAT had no obvious change between ESEP and EVOL group. In the PPI network (Supplementary Fig. 8), acetylcholine was associated with three up-regulated genes (PPFIA2, STX1A and RIMS1) and one down-regulated gene (BZRAP1) belonging to cholinergic anti-inflammatory pathway. These DEGs may play important roles in the pathogenesis of sepsis and may be relevant to its therapy and prognosis. Therefore, the elderly might differ from the young in cholinergic anti-inflammatory pathway [35-38].

### Oxidative stress

Besides inflammatory responses, we observed significant decreases in the expression of genes encoding components of the mitochondrial respiratory chain in ECLP group. Mitochondrial dysfunction contributed to organ failure in septic patients, and often associated with increased ROS production by the organelle itself [39]. ROS are not only cytotoxic molecules but also signaling molecules regulating a wide variety of physiological processes [40]. For example, by regulating TLR-initiated signaling pathways, ROS modulate the production of pro-inflammatory cytokines, focusing on Nrf2 pathway. Nrf2 (a key transcription factor) regulates oxidative stress and inflammation by activating target genes that encode antioxidant molecules [41]. Under the normal status, Nrf2 localizes in the cytoplasm binding with Keap1, and is rapidly degraded by the ubiquitin-proteasome pathway. ROS could target the Nrf2/Keap1 complex, dissociating Nrf2 from Keap1. Stabilized Nrf2 then translocate to the nucleus and binds to the antioxidant response elements to activate transcription of Nrf2-targeted downstream genes such as HO-1, which provide effective protection against oxidative stress in injured tissues [42]. In this study, compared to ESham group, ECLP group showed a significantly increased protein expression of Keap1 in the cytosol, which was only slightly increased in YCLP group. The translocation of Nrf2 to the nucleus in sepsis could only increase protein levels of HO-1 in the young, endowing them higher cellular antioxidant defense ability.

**Figure 8.**
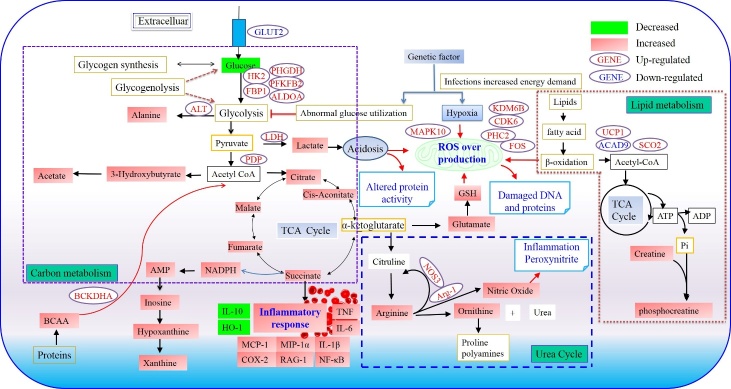
Hypothetical pathway constructed based on integration of gene-by-metabolite interactions Gene-by-metabolite interactions determined by average absolute value correlations for metabolomics families (e.g., TCA - succinate, fumarate, malate, citrate) to individual genes.

### ASCA analysis of age effects on septic rats

Sepsis is a severe immune inflammation response to bacterial infection. In the clinic, most elderly sepsis patients die of infection induced by immunosuppression or immune paralysis at a late stage of sepsis [1]. The metabolic profiles of the septic rats at different ages were compared to understand the discrepancies in their responses to sepsis (Supplementary Fig. 3). The factors of age and sepsis can both affect metabolic status. To resolve the individual effect of each factor on the outcomes, we performed an ASCA analysis (Fig. 5). Compared with YSham group, ESham group had elevated plasma lactate, betaine, and histamine levels. The YSham group were characterized by markedly increased allantoin, TMAO, and tyrosine levels relative to those of ESham group. Allantoin and TMAO levels were also increased in the ECLP group; however, no increase was observed in the YCLP group. Allantoin, widely used as a skin care agent, has anti-inflammatory, endotoxin-binding and cell-growth-promoting activity [43]. Its increase in sham rats suggested that those rats had stronger regeneration capacity than the middle-aged rats, resulting in more effective self-repair after injury. In addition, serum or plasma allantoin has been previously reported as a marker of free radical activity [44, 45]. As allantoin is the first and major product of uric acid oxidation, increased levels of allantoin in ECLP compared with YCLP might also reflect age-dependent free radical production in sepsis.

ASCA analysis also suggests that circulating levels of TMAO increase with aging. TMAO is an oxidation product of trimethylamine, which is derived from choline by gut bacteria. The increased level of TMAO in ECLP group might be due to irregular disruption and endotoxin translocation in the intestinal environment. The gut microbiota functions as a living barrier against ingested noxious substances and liberates otherwise inaccessible nutrients for systemic absorption [46]. In YSham group, the gut microbiota is largely intact, leading to an increased level of TMAO. Septic rats display early endotoxemia 6 to 12 h after induced CLP, resulting in circulating endotoxin. It has been noted that bacterial translocation from the gut is a possible cause of this phenomenon. Bacterial translocation can be defined as the passage of viable bacteria from the lumen of the gastrointestinal tract into other organ systems via the mesenteric lymphatic system. Under non-septic circumstances, the gastrointestinal mucosa is considered nearly impermeable to the nonpathogenic endogenous flora [47]. However, enhanced bacterial translocation and intestinal permeability follow severe infection in rats. Potentially toxic metabolites may arise from alterations in gut microbiota in a range of disorders, suggesting that the usual symbiotic relationship between the gut microbiota and the host may turn abnormal in these conditions, thus exacerbating disease [48, 49].

The aging process has been shown to induce alterations in the composition, diversity and functional features of the gut microbiota, and these changes are associated with an aging-related decline in immune system functioning and inflammation control [50]. We found that ESEP group had significantly higher levels of plasma TMAO than YCLP group, indicating that aging elevated circulating TMAO levels. The expression of pro-inflammatory cytokines, the activity of NO, and the level of superoxide production were also increased in ESEP groups. High TMAO levels have been reported to activate the well-known MAPK and NF-κB signaling cascades and account for vascular inflammation and oxidative stress (Fig. 8).

## Supplemetary Materials

The Supplemenantry data can be found online at: www.aginganddisease.org/EN/10.14336/AD.2018.1027.


