# Supplementary material for: Metabolomics Coupled with Transcriptomics Approach Deciphering Age Relevance in Sepsis
Source: Aging Dis. 2019 Aug 1;10(4):854–70. doi: 10.14336/AD.2018.1027 (PMC6675524; doi:10.14336/AD.2018.1027)
Supplement: Supplementary file 1 [file AD-10-4-854-s.pdf]

SUPPLEMENTARY DATA

Metabolomics Coupled with Transcriptomics Approach Deciphering Age Relevance in Sepsis

Dingqiao Xu[1], Shanting Liao[1], Pei Li[1], Qian Zhang[1], Yan Lv[1], Xiaowei Fu[1], Minghua Yang[1], Junsong Wang[2,*], Lingyi Kong[1,*]

[1]Jiangsu Key Laboratory of Bioactive Natural Product Research and State Key Laboratory of Natural Medicines, School of Traditional Chinese Pharmacy, China Pharmaceutical University, Nanjing, China
[2]Center for Molecular Metabolism, Nanjing University of Science and Technology, Nanjing, China.

© 2018. Xu D et al. Published online at http://www.aginganddisease.org/EN/10.14336/AD.2018.1026

SUPPLEMENTARY DATA

Animal model

After 12 h of fasting, rats were anesthetized with pentobarbital (60 mg·kg^{-1} body weight). The intestines were carefully exposed with a 2-3 cm ventral midline incision, and the cecum was carefully externalized. The distal 30% was ligated with a 2-0 silk suture to avoid intestinal obstruction and then punctured twice using a 16-gauge needle. The punctured cecum was squeezed to expel a small amount of fecal material and was returned to the abdominal cavity, after which the abdominal incision was closed in two layers. Sham-operated animals were subjected to laparotomy and intestinal manipulation; however, the cecum was not ligated or perforated. All animals received subcutaneous administration of normal saline (5 mL/100 g of body weight) immediately after the operation. Sham and CLP-operated rats were treated with equivalent volumes (10 mL·kg^{-1} bodyweight) of 0.5% CMC-Na only. The animals were monitored, and survival was recorded.

Sample preparation and ^{1}H NMR analysis

Serum protein was extracted in accordance with previously reported protocols [1]. Briefly, 300 μL serum samples were mixed with methanol in a 1:2 ratio (v/v), vortexed, and incubated at -20 °C for 20 min. The mixtures were centrifuged at RCF (reactive centrifugal force) = 13,400 × *g* for 30 min to pellet proteins. Supernatants were decanted into fresh vials and dried. The dried samples were dissolved in 600 μL of 99.8% D_2O phosphate buffer (0.2 mol L^{-1} Na_2HPO_4 and 0.2 mol L^{-1} NaH_2PO_4, pH 7.4, containing 0.05% TSP). TSP acted as a chemical shift reference (δ 0.0), D_2O provided a lock signal and phosphate buffer was added to minimize NMR shift variation due to the pH discrepancy. After vortexed and transferred to 5 mm NMR tubes. NMR spectra were recorded on a Bruker AVANCE III 500 MHz NMR spectrometer at 298 K. D_2O was used for field frequency locking, and TSP was used as a chemical shift reference (1H, 0.00 ppm). A transverse relaxation-edited Carr-Purcell-Meiboom-Gill (CPMG) sequence (90 (τ-180-τ) n-acquisition) with a total spin-echo delay (2 nτ) of 10 ms was used to suppress the signals of proteins. ^{1}H NMR spectra were measured with 128 scans for a total of 32,000 data points over a spectral width of 7,500 Hz. The spectra were Fourier transformed by multiplication of the FID (free induction decay) with an exponential weighting function corresponding to a line broadening of 0.5 Hz. With TopSpin software (version 3.0, Bruker Biospin, Germany), all the spectra were automatically phased, baseline corrected, and calibrated to TSP at 0.00 ppm. With MestReNova (Version 8.0.1, Mestrelab Research SL, Santiago de Compostela, Spain), ^{1}H NMR spectra were converted into ASCII files and aligned with a peak alignment graphical application (Pre-processing/Peak Alignment) in “R”, a freely available, open-source software (“R” Development Core Team, http://cran.r-project.org/), for further processing, and regions between 0.7 and 9.4 ppm were binned using an adaptive binning approach based on the code and removal of signals from water and its neighboring regions (approximately 4.6-5.58 ppm for plasma). The binned data were subjected to probabilistic quotient normalization, mean centered and Pareto scaled in “R” prior to multivariate statistical analysis.

Plasma sample preparation and analysis by GC/MS

A 100 μL aliquot of plasma sample was spiked with internal standard (15 μL heptadecanoic acid in water, 1 mg/mL) and vortexed for 2 min. The mixed solution was extracted with 400 μL methanol and centrifuged for 10 mins at a rotation speed of 13,000 rpm at 4 °C. An aliquot of 450 μL of the supernatant obtained was transferred to a clean glass sampling vial and dried under a gentle stream of nitrogen at room temperature. The residue derivation involved the addition of 80 μL methoxamine hydrochloride (20 mg/mL in pyridine), was added to the vial and kept at 60 °C for 4 h followed by the addition of 60 μL of MSTFA (1% TMCS) for 1 h at 70°C. Each 0.2 μL aliquot of the derivatized solution was injected in spitless mode into an Agilent 7890B gas chromatography coupled with an Agilent 5977A mass spectrometer. Separation was achieved on a HP-5MS capillary column (30 m × 0.25 mm I.D., 0.25-μm film thickness; Agilent J & W Scientific, Folsom, CA, USA) with helium as the carrier gas at a constant flow rate of 0.7 mL/min. The solvent delay time was set to 5 min. The temperature of injection, transfer interface, and ion source were set at 270 °C, 290°C, and 230 °C, respectively. The GC temperature programming was set to 2 min isothermal heating at 80 °C, followed by 10 °C/min oven temperature ramps to 300 °C, and a final 6 min maintenance at 300 °C. Electron impact ionization (70 eV) at full scan mode (m/z 30-600) was used. GC/MS metabolites were identified by comparing the mass fragmentations with NSIT 05 Standard mass spectral database in NIST MS search 2.0 (NIST, Gaithersburg, MD) software with a similarity of more than 70% and finally verified by available reference standards [2].

Quantification of gene expressions by real-time RT-PCR

© 2018. Xu D et al. Published online at http://www.aginganddisease.org/EN/10.14336/AD.2018.1026

SUPPLEMENTARY DATA

Real-time RT-PCR was performed to validate the results derived from RNA-seq. Total RNA from the blood of elderly rats and that of humans was isolated using TRIzol Reagent (Invitrogen). In addition, the quantity and quality of the RNA were determined using a SpectraMax Plus 384 enzyme-labeling instruments. Reverse transcription reactions were performed using 2 μL of total RNA and 4 μL of Prime Script RT Master Mix (Perfect Real Time) in a 20 μL mixture incubated for 15 min at 42 °C, followed by 5 min at 92 °C. The resulting cDNA was stored at -20 °C. Semi-quantitative PCR was performed using 2 μL of cDNA, 4 μM of the gene-specific oligonucleotide primer, and 10 μL of QuantiFast™ SYBR Green PCR mix (Vazyme, Nanjing, China) in a final reaction volume of 20 μL of SYBR Green PCR Master Mix (Vazyme, Nanjing, China) with a LightCycler 480 instrument (Roche Molecular Biochemicals, Mannheim, Germany). PCR was conducted for 40 cycles of denaturation at 94°C for 1 min, annealing at 55 °C for 1 min, and extension at 72 °C for 1 min, followed by a final extension step at 72 °C for 10 min. All PCR primers were purchased from Tongyong Technologies (Chuzhou, Anhui). The primers used in this study are listed in Supplemental Material (Table S6, S7). The ΔCt (cycle threshold) method was used for calculation of relative differences in mRNA abundance with the LightCycler 480. Data were normalized to the expression of β-actin mRNA and presented as fold change relative to the untreated control. The relative expression levels were calculated by comparing the Ct values of the target genes with that of β-actin using the $2^{-\Delta\Delta Ct}$ method [3].

Western blot analysis

For protein analysis, liver tissues were removed from aged rats and frozen until use. In preparation for the assay, they were rapidly thawed, mechanically homogenized and lysed for 30 min in 1x RIPA lysis buffer (50 mM Tris-HCl, pH 7.4, 150 mM NaCl, 0.25% deoxycholic acid, 1% NP-40, and 1 mM EDTA, Sigma-Aldrich, St. Louis, USA) containing 1% phosphatase and protease inhibitor. The lysates were centrifuged at 15,000 × *g* for 15 min at 4 °C, and the supernatants were kept at -78 °C. Protein concentrations were measured using a bicinchoninic acid (BCA) protein assay reagent kit (Beyotime, Haimen, China). The proteins were denatured at 95 °C for 10 min in loading buffer. For iNOS, p-tyrosine, Nrf2 and cleaved PARP analysis, 40 μg of protein was subjected to 8% polyacrylamide gel electrophoresis (PAGE) with sodium dodecyl sulfate (SDS) and transferred to polyvinyl difluoride (PVDF) membranes (Bio-Rad Inc., Hercules, CA, USA). For CD14, TLR4, TARF6, p-IKKα/β, p-p65, p-ERK, p-JNK, p-p38, Keap1, c-Jun and c-Fos detection, 60 μg of protein was subjected to 10% SDS-PAGE and transferred to PVDF. For MyD88, HMGB-1, HO-1, histone, Arg-1 and p-IκBα analysis, 30 μg of protein was subjected to 12% SDS-PAGE and transferred to PVDF. Nonspecific binding was blocked using 4% milk in TBST (TBS with 0.1% TWEEN 20), after which the membranes were incubated with the specific primary antibodies and HRP-conjugated secondary antibody. After extensive washing with TBST, antibody binding was visualized using a ChemiDOC XRS+ system (Bio-Rad, Inc.). The protein expression was quantified by use of Image Lab 4.0 (Bio-Rad, Inc., Hercules, CA).

References

[1] Fan W M (1996). Metabolite profiling by one-and two-dimensional NMR analysis of complex mixtures. Prog Nucl Mag Res Sp, 28:161-219.

[2] Constantinou C (2011). GC-MS Metabolomic analysis reveals significant alterations in cerebellar metabolic physiology in a mouse model of adult onset hypothyroidism. J Proteome Res, 10:869-879.

[3] Michael W, Pfaffl MH (2001). Validities of mRNA quantification using recombinant RNA and recombinant DNA external calibration curves in real-time RT-PCR. Biotechnol Lett, 23: 275-282.

© 2018. Xu D et al. Published online at http://www.aginganddisease.org/EN/10.14336/AD.2018.1026

SUPPLEMENTARY DATA

Supplementary Table 1. Clinical characteristics of the aged patients with sepsis.

Samples	Sepsis	Non-septic	p Value for Trend
Sample No.	158	71	-
Age (years)	67.3 (59-81)	65.1 (62-78)	-
male	62 %	70%	-
Body mass index, kg/m^2	23.8±3.6	24.5±3.2	0.159
Laboratory data			
IL-6 ($pg\cdot mL^{-1}$)	147.81±33.69	24.57±1.77	<0.001
MCP-1 ($pg\cdot mL^{-1}$)	371.29±154.95	137.35±22.90	<0.001
MIP-1α ($pg\cdot mL^{-1}$)	277.82±121.40	20.00±2.19	<0.001
RAG-1 ($pg\cdot mL^{-1}$)	259.68±63.62	31.05±2.97	<0.001
LECT2 ($pg\cdot mL^{-1}$)	25.92±5.07	42.38±7.55	<0.001
CX3CR1 ($pg\cdot mL^{-1}$)	183.12±11.35	40.75±4.86	<0.001
LDH ($U\cdot L^{-1}$)	1,662.53±178.62	1,177.55±142.37	<0.001
ALT ($U\cdot L^{-1}$)	179.27±122.28	18.21±0.38	<0.001
AST ($U\cdot L^{-1}$)	246.78±28.20	28.52±2.37	<0.001
Cre ($\mu mol\cdot L^{-1}$)	604.93±97.07	128.00±15.96	<0.001
NO (μM)	174.49±70.20	30.63±5.65	<0.001
SOD ($U\cdot mL^{-1}$)	8.65±3.37	72.63±1.04	<0.001
GSH ($\mu moL\cdot L^{-1}$)	19.21±4.80	37.05±6.81	<0.001
GSSG ($\mu moL\cdot L^{-1}$)	23.81±4.50	82.47±13.30	<0.001
GSH/GSSG ($\mu moL\cdot L^{-1}$)	0.83±0.27	0.47±0.15	<0.001
ACHE ($\mu g\cdot mg^{-1}$)	83.77±11.48	85.82±13.22	0.781
ChAT ($U\cdot mL^{-1}$)	72.72±11.33	70.28±7.10	0.664

Supplementary Table 2. Assignment results of the identified metabolites in patients.

No.	Metabolite	Assignments	Chemical shift
1	Leucine	δCH_3, δCH_3, γCH, αCH	0.94(t), 0.96(t), 1.71(m), 3.74(m)
2	Isoleucine	δCH_3, γCH_3, αCH	0.93(t), 1.0(d), 1.46(m)
3	Valine	γCH_3, γCH_3	0.98(d), 1.03(d), 2.26(m), 3.60(d)
4	3-Hydroxybutyrate	γCH_3, βCH, αCH_2	1.20(d), 2.31(m), 2.41(m), 4.16(m)
5	Lactate	CH_3, CH	1.32(d), 4.11(q)
6	Alanine	βCH_3, αCH	1.47(d), 3.78(q)
7	Lysine	δCH_2	1.48(m), 1.73(m), 1.91(m), 3.03(t), 3.76(t)
8	Acetate	CH_3	1.91(s)
9	NAA	CH_3, CH_2, CH	2.03(s), 2.51(m), 2.7(m), 4.4(m)
10	Methionine	S-CH3, βCH_2, S-CH2, α-CH,	2.13(s), 2.16(m), 2.65(t), 3.86(t)
11	Glutamate	βCH_2, βCH_2, γCH_2, αCH	2.10(m), 2.14(m), 2.36(m), 2.50(m), 3.77(t)
12	Glutathione	S-CH_2, N-CH, N-CH_2, CH_2	2.14(m), 2.55(m), 2.95(m), 3.77(m), 4.56(t)
13	Succinate	CH_2	2.43(s)
14	Glutamine	βCH_2, γCH_2, αCH	2.46(m), 3.77(t)
15	Arginine	CH_2, CH_2, CH_2, CH	1.87 (m), 1.90 (m), 3.23 (t), 3.76(t)
16	Creatine/ PCr	CH_2, CH_3	3.04(s), 3.93(s)
17	Ornithine	CH, CH2	3.06(t), 3.78(t)
18	Ethanolamine	O-CH, NH_2-CH_2,	3.13(d), 3.81(d)
19	Choline	$N(CH_3)_3$, N-CH_2	3.25(s), 3.51(m)
20	OPC	$N(CH_3)_3$, N-CH_2, O-CH_2	3.21(s), 3.57(t), 4.16(t)

© 2018. Xu D et al. Published online at http://www.aginganddisease.org/EN/10.14336/AD.2018.1026

21	Glucose	CH, CH_2	3.65-3.92(m)
22	Taurine	NH_2-CH_2, SO_3-CH_2	3.25(t), 3.43(t)
23	Betaine	$N(CH_3)_3$, CH_2	3.27(s), 3.90(s)
24	Glycine	CH_2	3.57(s)
25	Maltose	CH, CH_2	5.40(m), 5.23(d), 3.96(t)
26	Ascorbate	CH_2, CH	3.74(d),3.76(d),4.03(m),4.52(d)

PCr, Phosphocreatine; OPC, O-Phosphocholine
s, singlet; d, double; t, triple; q, quartet; m, multiple; dd, double doublet

Supplementary Table 3. Summary of reads in EVOL and ESEP group in RNA-seq experiment.

Sample	Total reads	Total mapped reads	Mapping percentage
EVOL	52,282,764	47,169,272	90.22%
EVOL	50,720,560	46,511,251	91.70%
EVOL	59,033,842	53,836,391	91.20%
ESEP	46,913,302	42,713,262	91.05%
ESEP	43,780,854	38,814,602	88.66%
ESEP	48,406,010	42,804,840	88.43%

Supplementary Table 4. Contributions to the total variation and p values of ASCA models by 1000 permutations test.

Submodel	Contribution to the total data variation (%)	P values
Factor1(age)	14.93	0.001
Factor2(CLP)	22.13	0.001
Interaction12	5.33	0.287
Residual	57.62	

© 2018. Xu D et al. Published online at http://www.aginganddisease.org/EN/10.14336/AD.2018.1026

SUPPLEMENTARY DATA

Supplementary Table 5. Putative identification of the metabolites corresponding to the ASCA procedure according to the factors for which they show a higher loading value.

NO.	Metabolite	Factors		
		Age	CLP	Age×CLP
1	Isoleucine	—	↑	—
2	Leucine	—	↑	↑
3	Valine	—	↑	—
4	3-Hydroxybutyrate	—	↑	—
5	Lactate	↓	↑	↑
8	Acetate	↑	↑	↑
10	Methionine	—	↑	—
13	Succinate	↑	↑	—
16	Creatine/Pcr	↑	↑	↑
21	Glucose	↓	↓	↑
31	Tyrosine	↑	↑	↑
32	Histamine	—	↓	↑
33	Tryptophan	↑	↑	↑
34	Phenylalanine	↑	↑	—
36	3-Methylxanthine	—	↑	↑
	TMAO	↑	↓	↓
	Allanion	↑	—	↑

"—": Not detected; "↑": Increased; "↓": Decreased.

.

Supplementary Table 6. List of the primers of patients used in this study.

Primer name	Forward primer	Reverse primer
TNF-α	TATCCTGGGGGACCCAATGT	AGCTTCTTCCCACCCACAAG
CD14	ACGATGAAGATTTCCGCTGC	ATCGACGCGCTTTAGAAACG
IL-6	GCGATGGAGTCAGAGGAAACTC	TCGCAAGACACAACTAGGGG
IL-10	TCCATTAGTGGCATGCTCATTC	TCCAATTGACATGGTTTGCAAG
CXCL13	TGGACCCTCAAGCTGAATGGA	CTGCCCAGGAAAGGGAAACTC
CXCL3	TGTGAATGTAAGGTCCCCCG	TTTCTGAACCATGGGGGATG
Arg1	TCCAAGGTCTGTGGGAAAAGC	TCCAATTGCCAAACTGTGGTC
Btla	ATCCAGGCTCTTCCTACTCTCC	GCTTGCCATTTCGTCCTTGG
HIF1α	TGTCTCCATTACCCACCGCTGA	GATCCTGAATCTGGGGCATGGT
Trem1	GCGTCCGAATGGTCAACCTT	TCTCATTGGAGCCAGGGGTC

© 2018. Xu D et al. Published online at http://www.aginganddisease.org/EN/10.14336/AD.2018.1026

SUPPLEMENTARY DATA

Supplementary Table 7. List of the primers of rats used in this study.

Primer name	Forward primer	Reverse primer
TNF-α	GCCCTACGGGTCATTGAGAG	TTGTTCCACAGGGGTCTTGG
CD14	CAGACACACCAGAAGAGGGCA	GAAAGAGAGCTGGAGGGATGG
IL-6	GCTAAGGACCAAGACCATCCAA	TGACCACAGTGAGGAATGTCCA
IL-10	TGCCAAGCCTTGTCAGAAATG	CTCCCAGGGAATTCAAATGCT
CXCL13	TTCCCTCTACAAACACGGCTG	GCCTGCTCGAAAATATCCTCC
CXCL3	GCTCCCAGGCTTCAGAAAATC	GGGATCGACTCGGACGTTATT
Arg1	CCTGCATATCTGCCAAGGACA	CATCACTTTGCCAATTCCCAG
Btla	CCTAAAGGGAACCACACACCC	GCAGCCTTAAGCCAGTCCATC
HIF1α	AGGATTCCAGCAGACCCAGTT	TGGGTAGAAGGTGGAGATGCA
Trem1	CACTCAACTCCAACCCGATCC	GATGAGGAGCCCACAGACCAC

© 2018. Xu D et al. Published online at http://www.aginganddisease.org/EN/10.14336/AD.2018.1026

SUPPLEMENTARY DATA

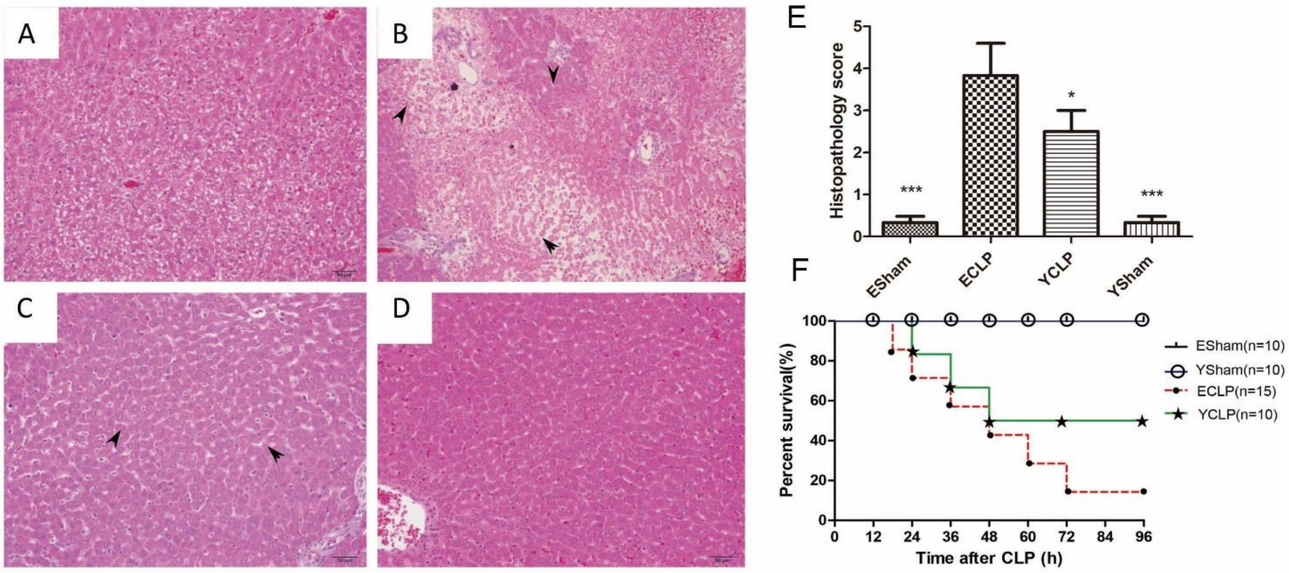

Supplementary Figure 1. Histological micrographs of liver. Tissues stained with hematoxylin and eosin (original magnification 200×): ESham **(A),** ECLP **(B),** YCLP **(C),** and YSham **(D).** Representative images (**E**) were chosen from the different experiment groups. YSham operations were performed as a control. (F) The result of the survival experiments. All animals were sacrificed 24 h after CLP. The survival rate was 100% for all ESham and YSham animals.

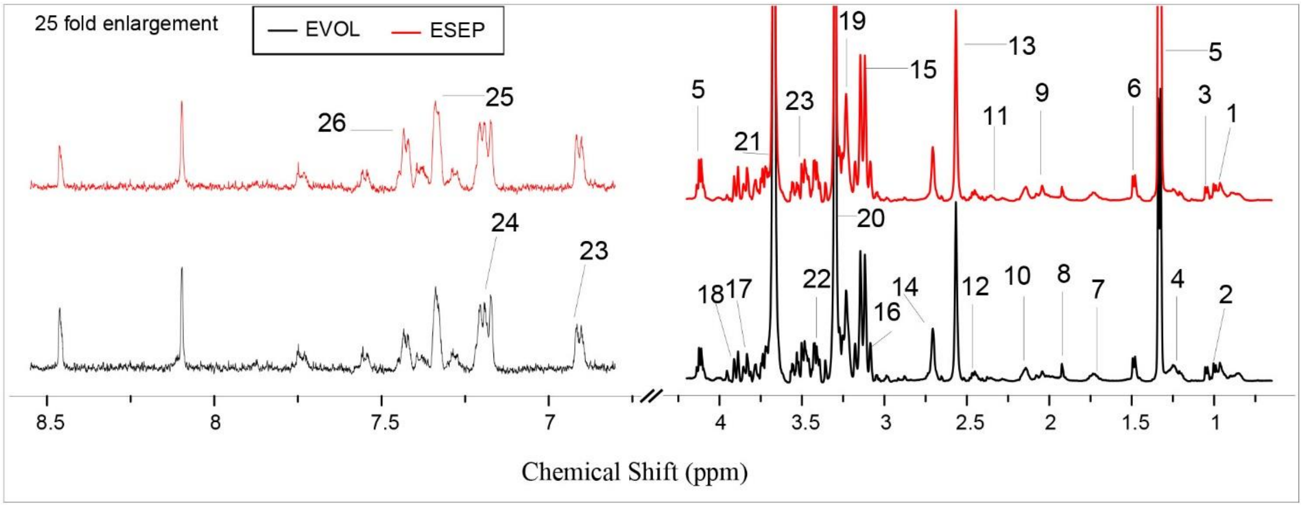

Supplementary Figure 2. Typical 500 MHz 1H NMR spectra of plasma with the metabolites labeled. Metabolites: 1, Leucine; 2, Isoleucine; 3, Valine; 4, 3-Hydroxybutyrate; 5, Lactate; 6, Alanine; 7, Lysine; 8, Acetate; 9, NAA; 10, Methionine; 11, Glutamate; 12, Glutathione; 13, Succinate; 14, Glutamine; 15, Arginine; 16 ,Creatine/PCr; 17, Ornithine; 18, Ethanolamine; 19, Choline; 20, OPC; 21, Glucose; 22, Taurine; 23, Betaine; 24, Glycine; 25, Maltose; 26, Ascorbate.

© 2018. Xu D et al. Published online at http://www.aginganddisease.org/EN/10.14336/AD.2018.1026

SUPPLEMENTARY DATA

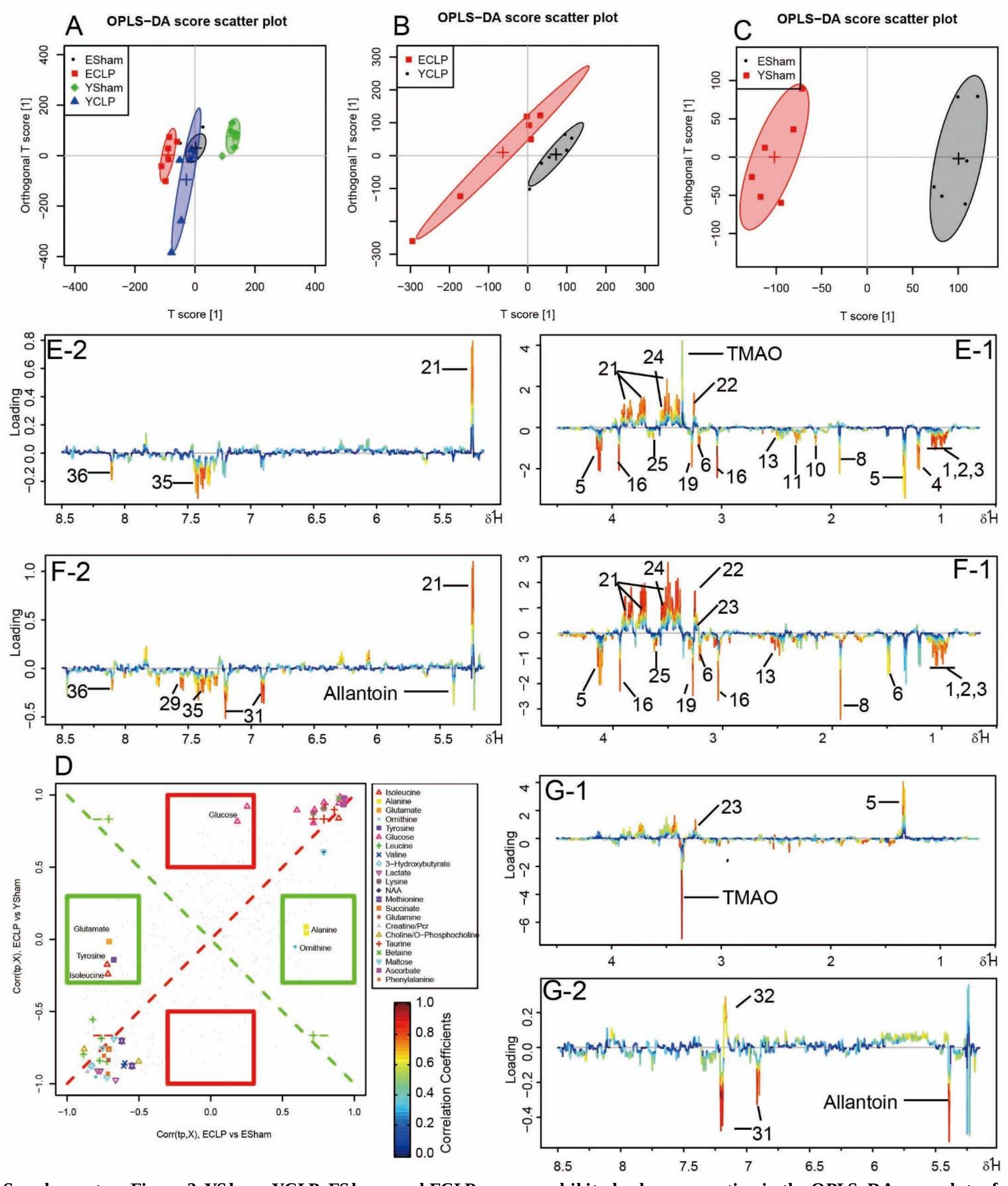

Supplementary Figure 3. YSham, YCLP, ESham, and ECLP groups exhibited a clear separation in the OPLS-DA score plots of plasma extracts. The color-coded loading plots revealed the differential plasma metabolites between groups. Comparisons of ECLP vs. YCLP and ESham vs. YSham were showed in 3B, C and 3F, G, respectively.

© 2018. Xu D et al. Published online at http://www.aginganddisease.org/EN/10.14336/AD.2018.1026

SUPPLEMENTARY DATA

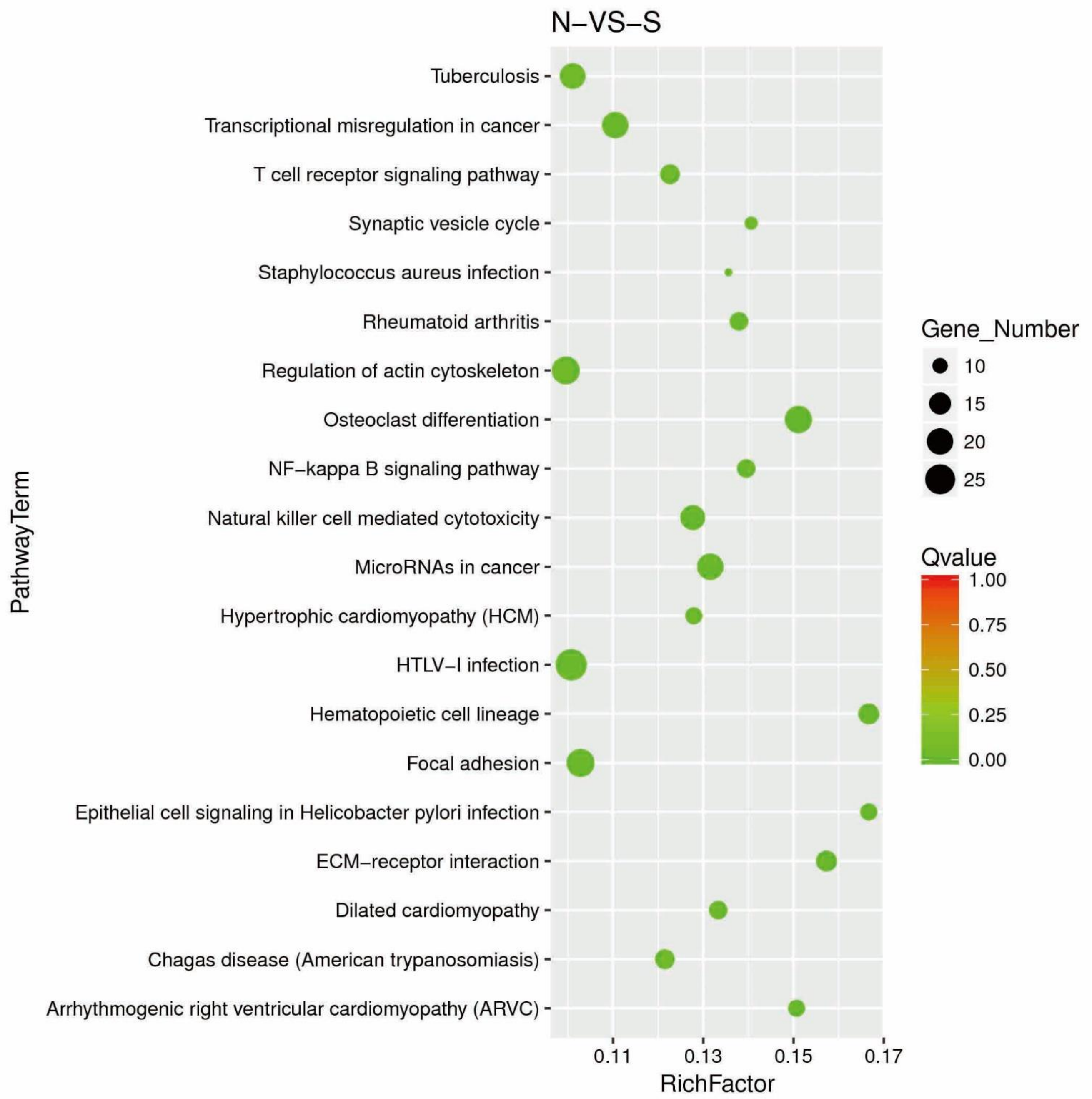

Supplementary Figure 4. Scatter diagram of KEGG enrichment analysis shows the 20 most significantly enriched pathways in elderly septic patients (ESEP). KEGG ontology assignments were used to classify the functional annotations of the identified genes to further understand their biological functions.

© 2018. Xu D et al. Published online at http://www.aginganddisease.org/EN/10.14336/AD.2018.1026

SUPPLEMENTARY DATA

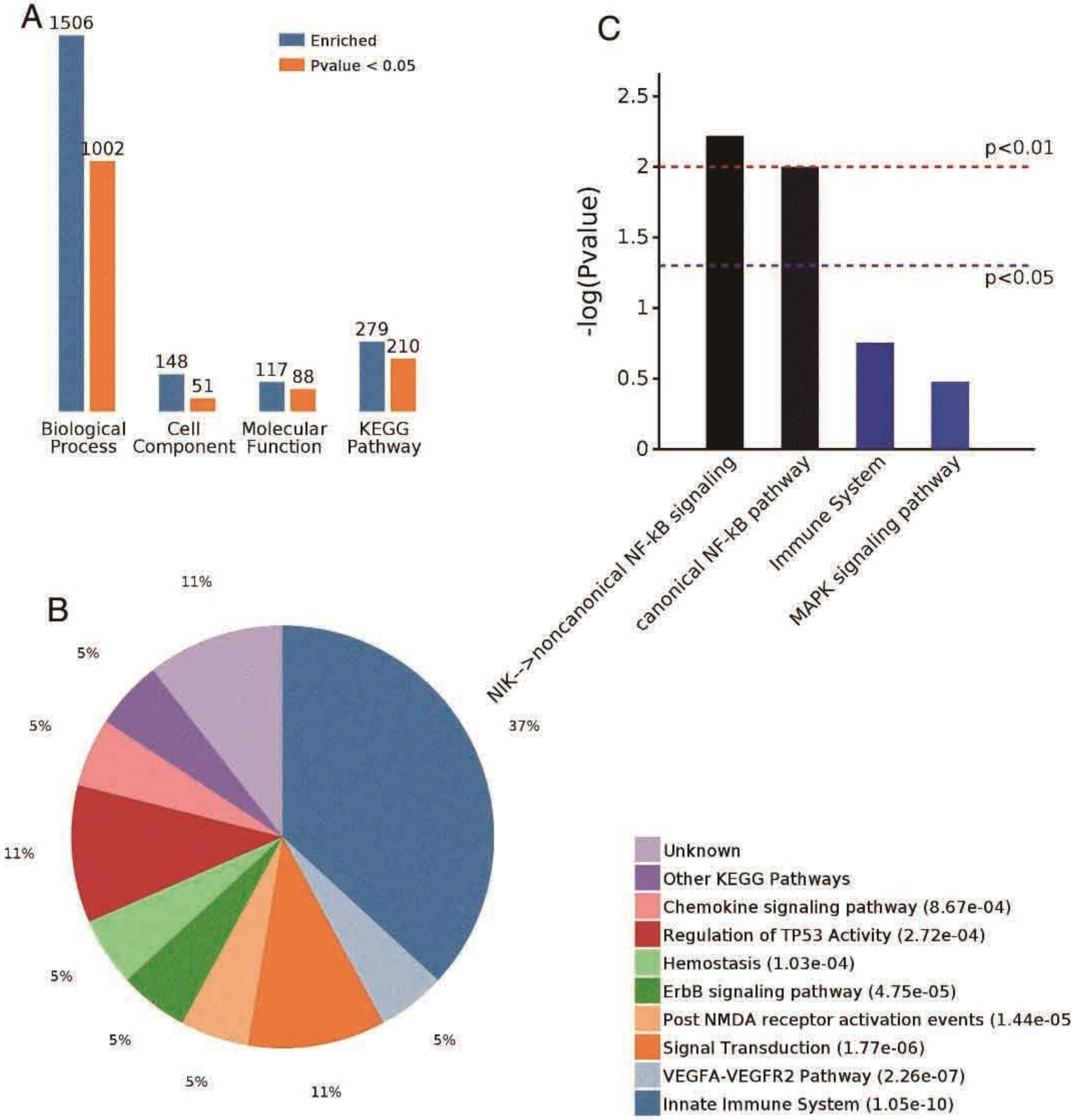

Supplementary Figure 5. Biological pathway analysis reveals dysregulation in immune and inflammation response pathways. (A) GO annotation of identified sepsis-related gene in three categories: biological process (BP), cellular component (CC) and molecular function (MF). **(B)** Distribution of enriched KEGG pathway. Columns refer to related pathways, which are colored with gradient colors from midnight blue (smaller p-value) to lighter blue (bigger p-value). **(C)** Sepsis activated several important pathways, such as Toll-like receptor signaling, and the canonical NF-κB, immune and the MAPK signaling pathways.

© 2018. Xu D et al. Published online at http://www.aginganddisease.org/EN/10.14336/AD.2018.1026

SUPPLEMENTARY DATA

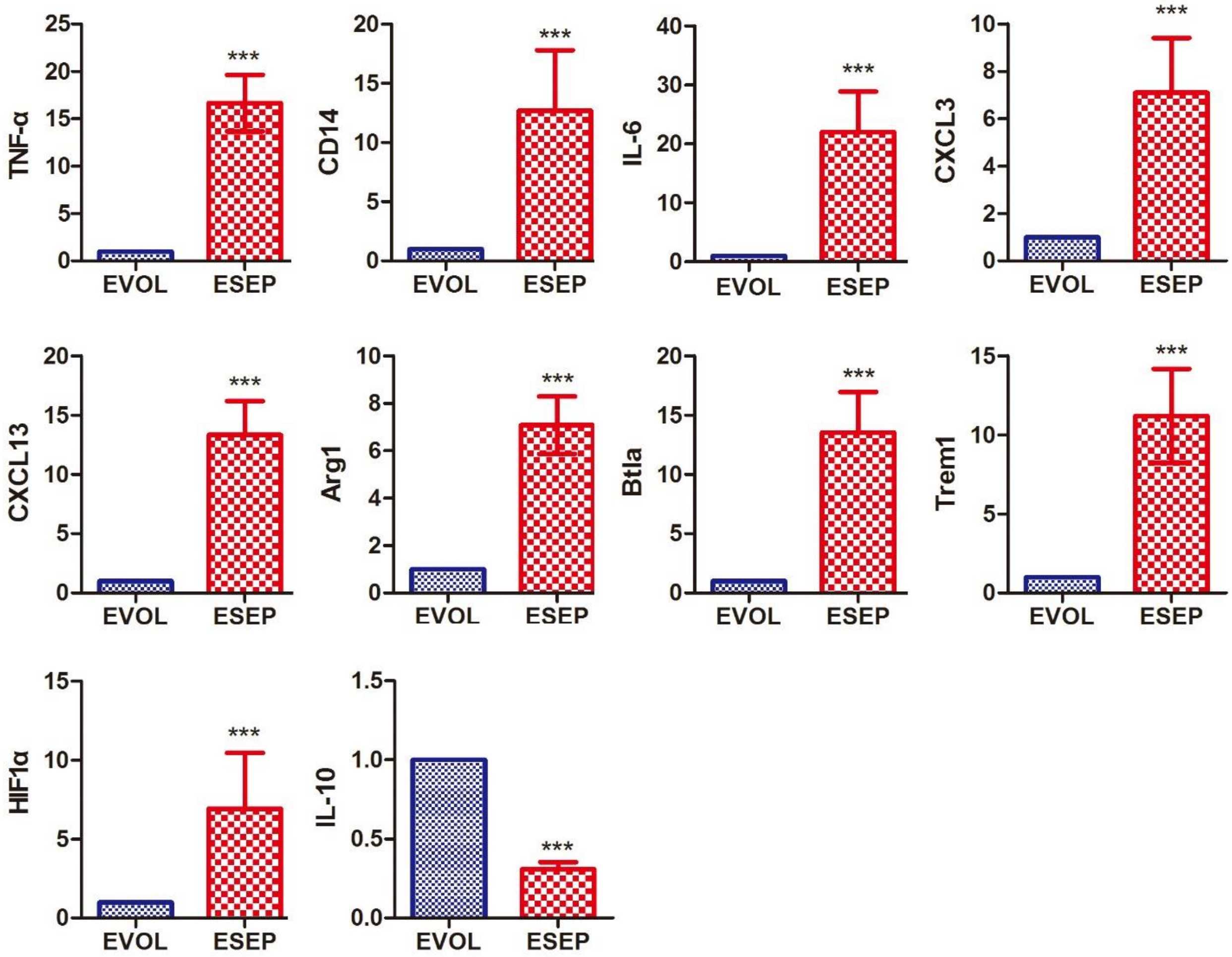

Supplementary Figure 6. Relative mRNA levels of cytokines were measured by qRT-PCR in plasma of elderly septic patients (ESEP). Histograms for plasma levels of TNF-α, CD14, IL-6, CXCL13, Arg1, CXCL3, Btla, HIF1α and Trem1 in each group. Data in plasma are expressed mean ± SD. *p < 0.05 and ***p < 0.001 for EVOL group vs. ESEP group. (n=8).

© 2018. Xu D et al. Published online at http://www.aginganddisease.org/EN/10.14336/AD.2018.1026

SUPPLEMENTARY DATA

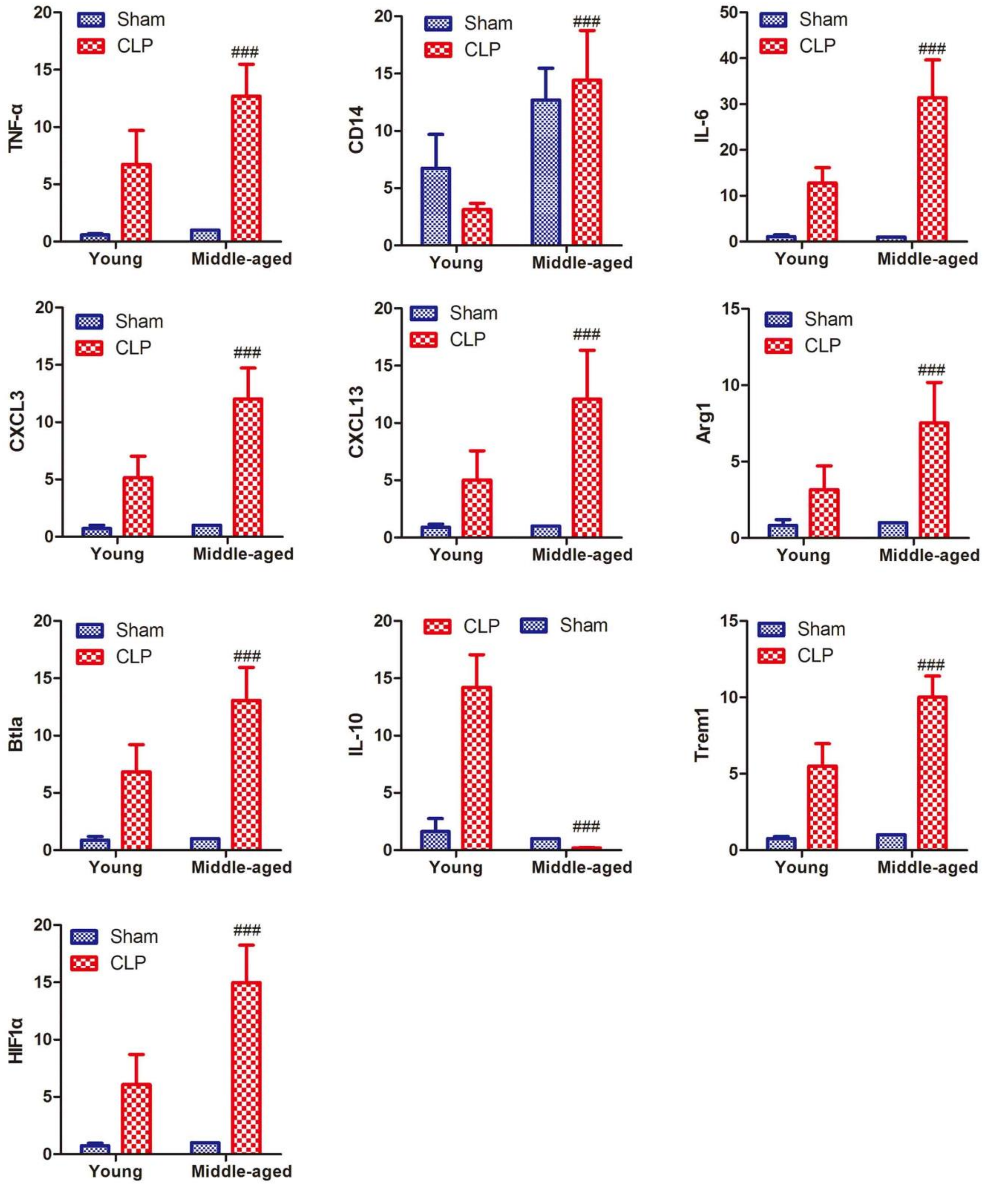

Supplementary Figure 7. Relative mRNA levels of cytokines were measured by qRT-PCR in plasma of middle-aged (ECLP) and young septic rats (YCLP) septic rats. Boxplots for plasma levels of TNF-α, CD14, IL-6, CXCL13, Arg1, CXCL3, Btla, HIF1α and Trem1 in each group. Data in plasma are expressed mean ± SD. #p < 0.05 and ###p < 0.001 for ECLP group vs. YCLP group. (n=8).

© 2018. Xu D et al. Published online at http://www.aginganddisease.org/EN/10.14336/AD.2018.1026

SUPPLEMENTARY DATA

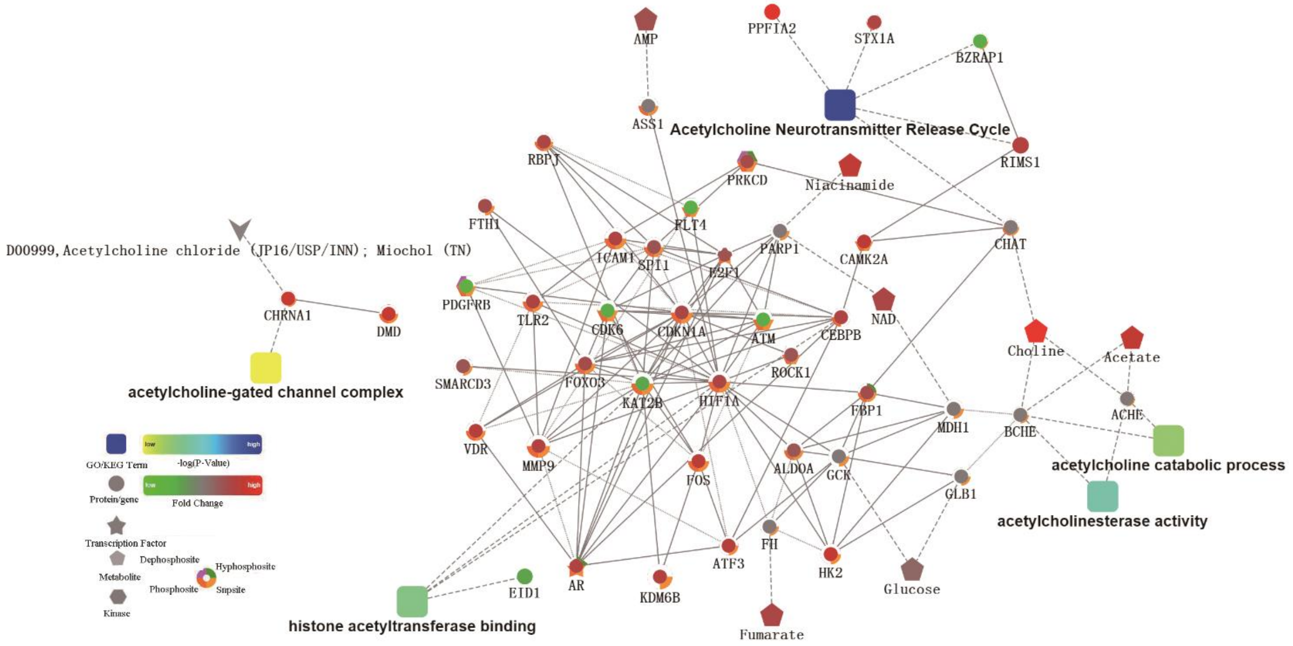

Supplementary Figure 8. A network of protein-protein interaction (PPI). The PPI analysis was based on fold change of gene/protein, protein-protein interaction, KEGG pathway enrichment and biological process enrichment. Circle nodes refer to genes/proteins. Rectangle refers to KEGG pathway or biological process, which was colored with gradient color from yellow (smaller p-value) to blue (bigger p-value).

© 2018. Xu D et al. Published online at http://www.aginganddisease.org/EN/10.14336/AD.2018.1026
